# Circulating cell-free methylated DNA reveals tissue-specific, cellular damage from radiation treatment

**DOI:** 10.1172/jci.insight.156529

**Published:** 2023-07-24

**Authors:** Megan E. McNamara, Netanel Loyfer, Amber J. Kiliti, Marcel O. Schmidt, Sapir Shabi-Porat, Sidharth S. Jain, Sarah Martinez Roth, A. Patrick McDeed, Nesreen Shahrour, Elizabeth Ballew, Yun-Tien Lin, Heng-Hong Li, Anne Deslattes Mays, Sonali Rudra, Anna T. Riegel, Keith Unger, Tommy Kaplan, Anton Wellstein

**Affiliations:** 1 Lombardi Comprehensive Cancer Center, Georgetown University Medical Center, Washington DC, USA.; 2 School of Computer Science and Engineering, The Hebrew University of Jerusalem, Jerusalem, Israel.; 3 Medstar Georgetown University Hospital, Washington DC, USA.; 4 Science and Technology Consulting, LCC, Farmington, Connecticut, USA.; 5 Faculty of Medicine, The Hebrew University of Jerusalem, Jerusalem, Israel.

**Keywords:** Genetics, Oncology, Breast cancer, Epigenetics, Radiation therapy

## Abstract

Radiation therapy is an effective cancer treatment, although damage to healthy tissues is common. Here we analyzed cell-free, methylated DNA released from dying cells into the circulation to evaluate radiation-induced cellular damage in different tissues. To map the circulating DNA fragments to human and mouse tissues, we established sequencing-based, cell-type-specific reference DNA methylation atlases. We found that cell-type-specific DNA blocks were mostly hypomethylated and located within signature genes of cellular identity. Cell-free DNA fragments were captured from serum samples by hybridization to CpG-rich DNA panels and mapped to the DNA methylation atlases. In a mouse model, thoracic radiation-induced tissue damage was reflected by dose-dependent increases in lung endothelial and cardiomyocyte methylated DNA in serum. The analysis of serum samples from patients with breast cancer undergoing radiation treatment revealed distinct dose-dependent and tissue-specific epithelial and endothelial responses to radiation across multiple organs. Strikingly, patients treated for right-sided breast cancers also showed increased hepatocyte and liver endothelial DNA in the circulation, indicating the impact on liver tissues. Thus, changes in cell-free methylated DNA can uncover cell-type-specific effects of radiation and provide a readout of the biologically effective radiation dose received by healthy tissues.

## Graphical abstract

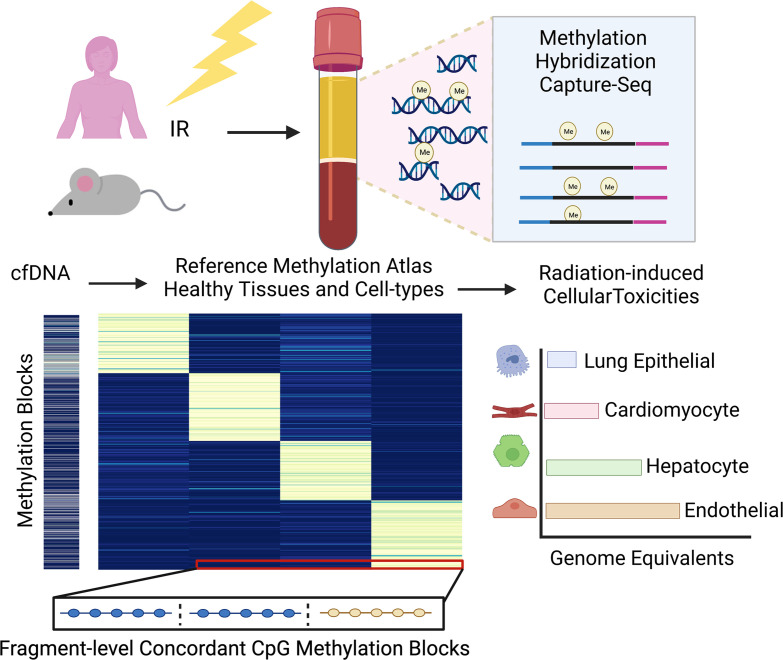

## Introduction

Radiation therapy is an effective cancer treatment; however, surrounding normal tissues are also affected, leading to tissue damage and remodeling (1–4). For patients with breast cancer, the heart, lungs, and skin are the most common organs at risk for toxicity (5–7). Onset of radiation-induced toxicities often vary due to patient-specific factors, and clinical symptoms may be acute or long-term, often appearing months or even years after treatment (6). Modern advances in radiation therapy now allow for administration of higher doses to smaller volumes, making target coverage and dose delivery important to maintain efficacy while minimizing toxicity. However, there are few biomarkers of radiation-related damage that allow for real-time monitoring of the tissue impact at a cellular level following radiation and provide a comparison with the planned dose. Here, we address this unmet need for sensitive and tissue-specific detection of cellular injury using serially collected blood samples.

Decoding the cellular origins of circulating cell-free DNA (cfDNA) from blood samples (liquid biopsies) is a promising approach for noninvasive monitoring of organ homeostasis, where rising levels of cfDNA released from dying cells indicate increased tissue damage (8–13). The majority of cfDNA fragments peak around 167 bp, corresponding to the length of DNA wrapped around a nucleosome (147 bp) plus a linker fragment (20 bp). This nucleosomal footprint in cfDNA reflects degradation by nucleases as a by-product of cell death and the tissue origins of the cfDNA fragments can be uncovered using highly cell-type-specific DNA methylation patterns (8, 14). DNA methylation typically involves covalent addition of a methyl group to the 5-carbon of cytosine (5mc), with the human and mouse genomes containing 28 and 21 million CpG sites, respectively (14, 15). Dynamic changes to the methylome during development and cellular differentiation lead to stable, cell-type-specific patterns of DNA methylation that are conserved during DNA replication and thus provide the predominant mechanism for inherited cellular memory during cell growth (16–19).

Recent studies have demonstrated the feasibility of tissue-of-origin analysis of methylated cfDNA in the circulation (20–24). However, few of these studies have focused on tracking intervention-related changes over time that is feasible by analyses of serially collected liquid biopsies (25–27). The short half-life of cfDNA (15 minutes to 2 hours) is ideal for detecting real-time changes in tissue homeostasis due to therapeutic interventions (27, 28). Also, few cfDNA analyses have taken advantage of CpG pattern analysis to increase sensitivity and specificity of cell type proportion estimates (22, 23, 29–32). Each cfDNA molecule originates from a defined cell and pattern analysis of sequence reads allows for individual classification of each sequenced fragment, as opposed to traditional methods that average the methylation status across a population of fragments aligned at single CpG sites (27, 28). Building on these advances, we present a fine-tuned approach for deconvolution of cfDNA patterns based on fragment-level CpG methylation blocks.

Here, we first report comprehensive, sequencing-based DNA methylation reference maps of healthy human and mouse cell types and show the close relationship of DNA methylation with cellular gene expression. Then, we apply cell-type-specific DNA methylation to trace the origins of cfDNA in serum samples. We report that hybridization capture sequencing of methylated cfDNA in serum samples reveals dose-dependent tissue damages in a mouse model of radiation injury. In addition, analyses of serial serum samples from patients with breast cancer undergoing standard-of-care radiation treatment indicate distinct cellular damages in different organs and provide a measure of the biologically effective radiation dose administered. Thus, as a proof of concept, the atlases of cell-type-specific methylation signatures developed here allow for detection and quantification of radiation-induced cellular injury from cfDNA in the circulation.

## Results

### Experimental paradigm to identify the cellular origins of radiation-induced damage from cfDNA in the circulation.

**Figure 1:**
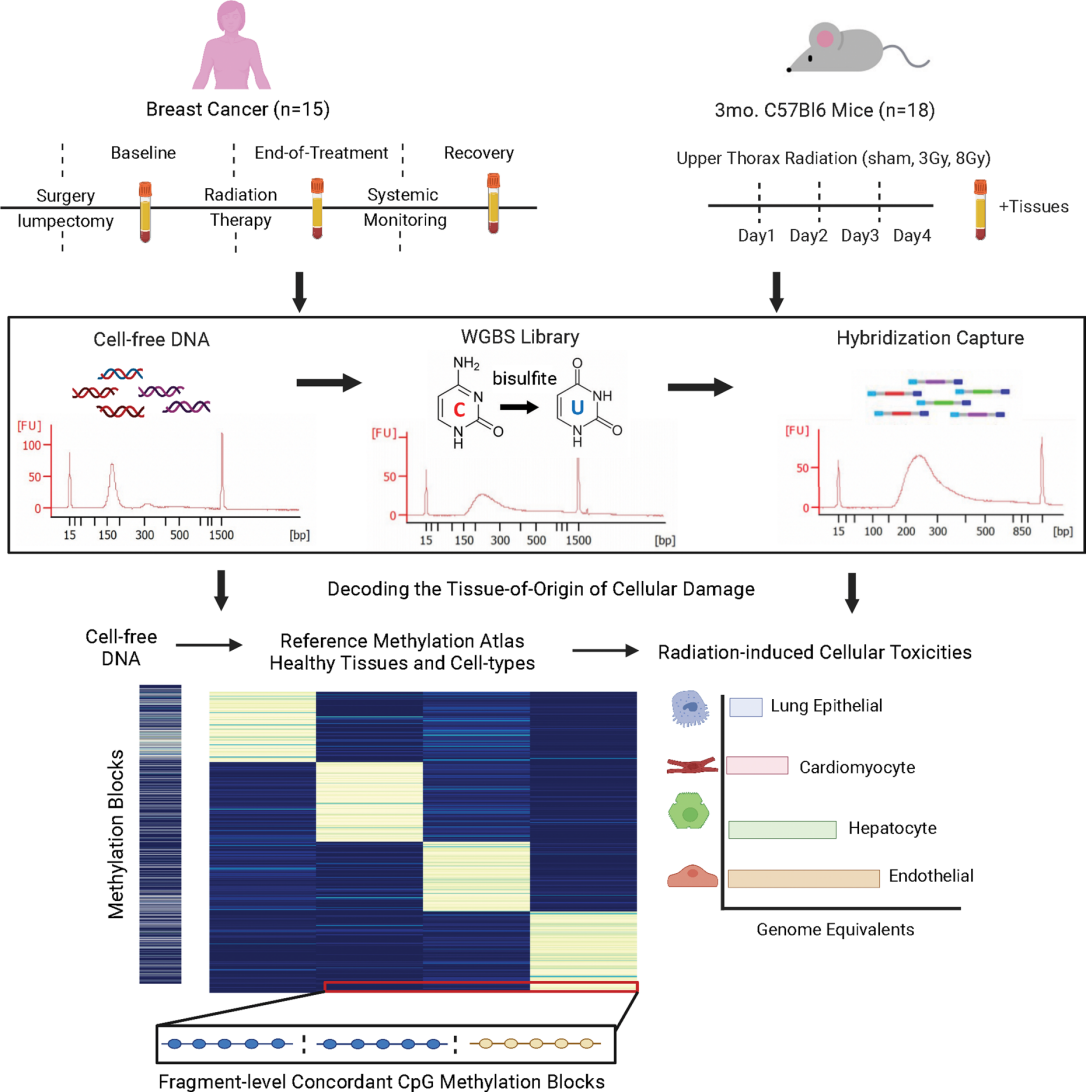
Experimental paradigm using cell-free methylated DNA in blood to identify cellular origins of radiation-induced tissue damage. Serial serum samples were collected from breast cancer patients treated with radiation. In parallel, paired serum and tissue samples were collected from mice receiving 3 Gy or 8 Gy of radiation compared to sham control. Cell-free DNA (cfDNA) methylome profiling of serum samples was performed using hybridization capture-sequencing of bisulfite-treated cfDNA. Cell-type-specific methylation blocks were identified from whole-genome bisulfite sequencing (WGBS) reference data of healthy tissues and used to identify the cellular origins of the serum cfDNA.

To investigate whether radiation-induced tissue damage can be monitored from changes in methylated cfDNA in the circulation, we collected serial serum samples from patients with breast cancer undergoing routine radiation treatment as well as serum and tissue samples from mice that had received different doses of thoracic radiation (Figure 1). The bioanalyzer trace in Figure 1 shows readings of cfDNA isolated from serum samples that were bisulfite treated, enriched for sequences of interest by methylome-wide hybridization capture, and subjected to sequence analysis. As a prerequisite for identification of the cellular origins of the cfDNA fragments isolated from the circulation, we established human and mouse cell-type-specific DNA methylation atlases. We took a sequencing-based approach interrogating existing whole-genome bisulfite sequencing (WGBS) data sets and generated complementary data from additional cell types composing at-risk organs that include the lungs, heart, and liver (Supplemental Tables 1 and 2; supplemental material available online with this article; https://doi.org/10.1172/jci.insight.156529DS1). The characteristics and validation of the DNA methylation blocks that provide the basis for the cell-type-specific mouse and human atlases are described next.

### Differences in DNA methylation blocks reflect distinct developmental lineages and cellular identities.

**Figure 2:**
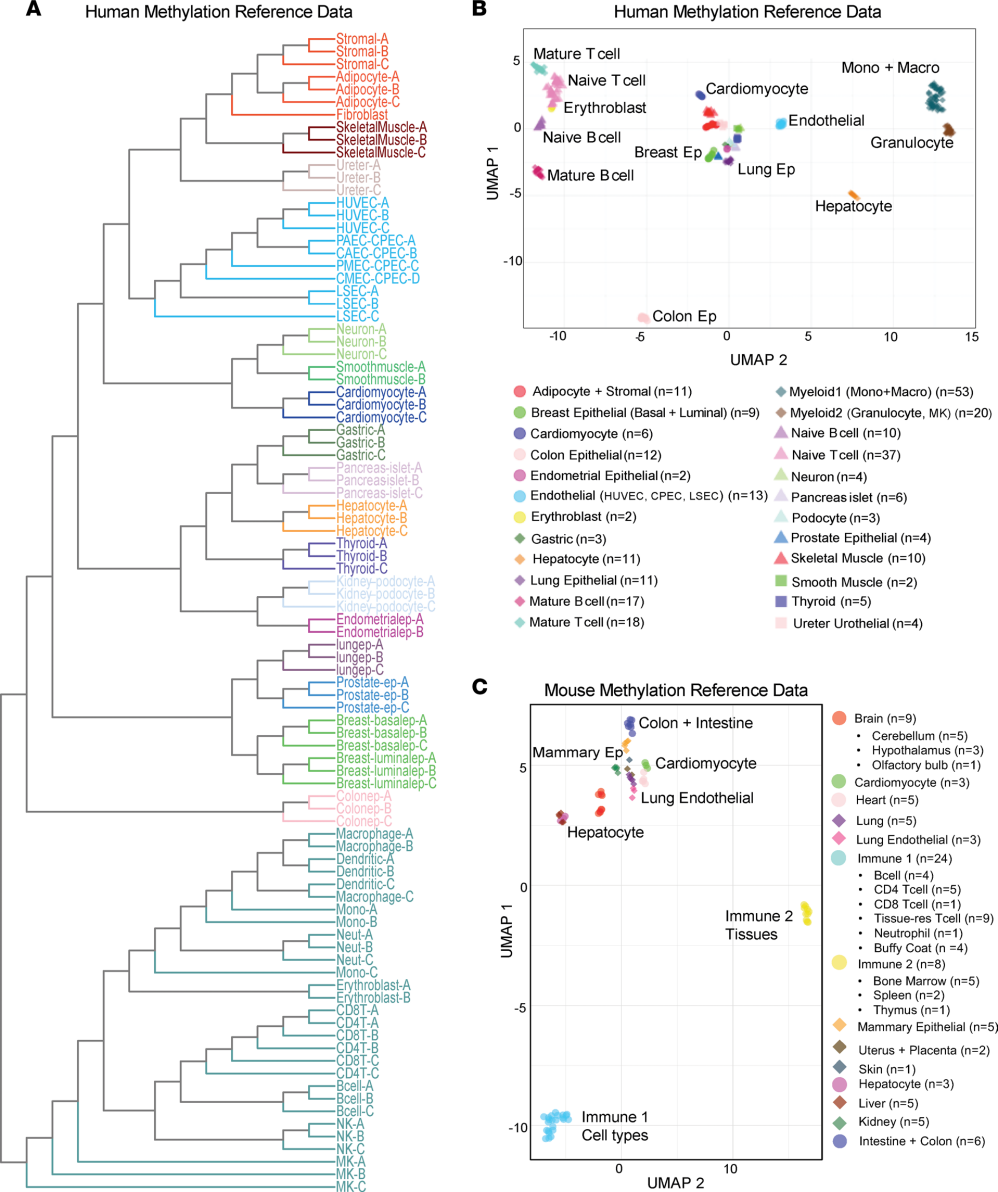
Characterization of human and mouse healthy cell–type-specific reference methylation data. (**A**) Tree dendrogram depicting the relationship between different cell types. Whole-genome bisulfite sequencing (WGBS) data sets were included in the analysis. Average methylation was calculated for each sample within blocks of at least 3 CpGs and the top 30,000 blocks were selected that showed the highest variability across all samples. Unsupervised clustering of the reference WGBS samples was performed based on similarity in methylation status at these highly variable blocks. Samples from cell types with greater than *n* = 3 replicates were merged. (**B** and **C**) UMAP plot of human (**B**) and mouse (**C**) WGBS reference data sets. CAEC, coronary artery endothelial cell; CMEC, cardiac microvascular endothelial cell; CPEC, joint cardiopulmonary endothelial cell; HUVEV, human umbilical vein endothelial cell; LSEC, liver sinusoidal endothelial cell; MK, megakaryocyte; NK, natural killer cell; PAEC, pulmonary artery endothelial cell; PMEC, pulmonary microvascular endothelial cell.

We obtained controlled access to reference human and mouse WGBS data sets from publicly available databases, preferentially from primary cells isolated from healthy human and mouse tissues. Additionally, we generated cell-type-specific methylomes for mouse immune cell types (CD19^+^ B cells, Gr1^+^ neutrophils, CD4^+^ T cells, and CD8^+^ T cells) and human tissue-specific endothelial cell types (coronary artery, pulmonary artery, cardiac microvascular, and liver sinusoidal endothelial cells [LSECs]). This resulted in the curation of mouse methylation data from 10 different cell types and 18 tissues to establish the most comprehensive mouse methylation atlas to date. In addition, we characterized methylation data from over 30 distinct human cell types from diverse populations of donors (Supplemental Tables 1 and 2). To better understand the epigenomic landscape of these healthy human and mouse cell types in tissues, we characterized the methylomes by first segmenting the data into homogeneously methylated blocks where DNA methylation levels at adjacent CpG sites are highly correlated across different cell types (22). Curated human WGBS data sets from healthy cell types were segmented to identify 351,395 blocks covered by our hybridization capture panel used in the analysis of cfDNA in human serum (captures 80 Mb, ~20% of CpGs). Likewise, segmentation of mouse WGBS data sets from healthy cell types and tissues identified 1,344,889 blocks covered by the mouse hybridization capture panel (captures 75 Mb, ~50% of CpGs). Average methylation was calculated within blocks of at least 3 CpG sites and the top 30,000 blocks (~10%) were selected, showing the highest variability across all samples. Unsupervised clustering analysis of the top 30,000 variable methylation blocks among all human samples revealed that cellular identity and developmental lineage primarily drives the relationship between samples and is presented as a dendrogram and uniform manifold approximation and projection (UMAP) plot in Figure 2, A and B. The respective analysis of mouse cell types is depicted in Figure 2C and Supplemental Figure 1A. The tight relationship of methylomes of the same cell type observed from the cluster analysis reinforces the concept that methylation status is conserved at regions critical to cell identity. The variation in distance between all samples was approximately 12 times larger than the variation in distance between samples from the same cell type (see Methods). It is noteworthy that differences in library construction did not bias sample clustering (Supplemental Figures 2 and 3). This stability allows methylated DNA to serve as a robust biomarker of cell types across diverse human populations. For the most part, cells from distinct lineages remain closely related, including immune, epithelial, neuronal, endothelial, muscle, and stromal cell types. Examples are tissue-specific endothelial and tissue-resident immune cells that cluster with other endothelial or immune cells respectively, independent of the germ layer origin of their tissues of residence. Collectively, these findings support the idea that DNA methylation is highly cell-type specific and reflects cell lineage specification.

### Development of sequencing-based DNA methylation atlases of primary human and mouse cell types.

**Figure 3:**
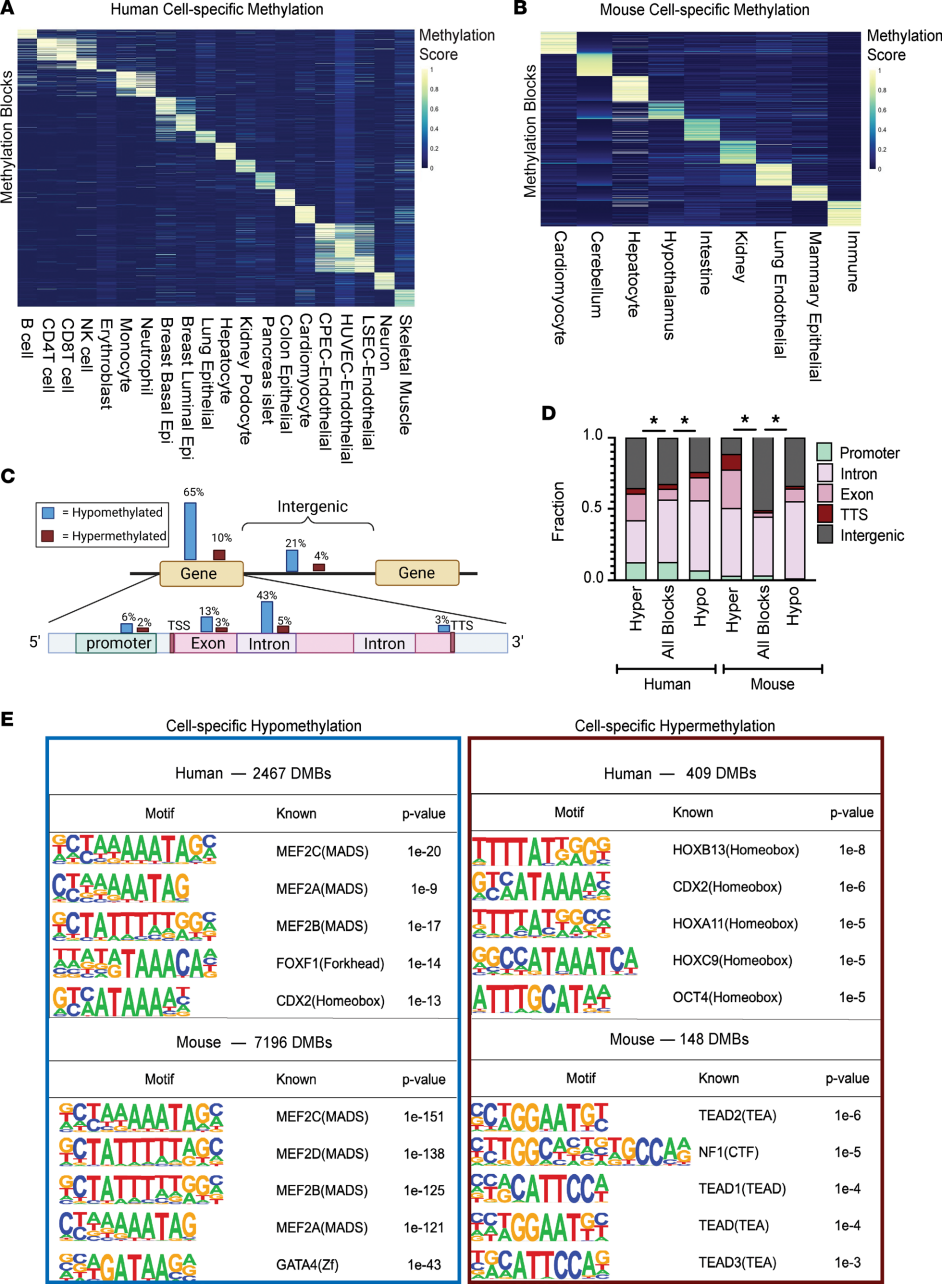
Cell-type-specific DNA blocks are mostly hypomethylated, enriched at intragenic regions and developmental transcription factor (TF) binding motifs. (**A** and **B**) Heatmaps of differentially methylated cell-type-specific blocks identified from reference WGBS data compiled from healthy cell types and tissues in human (**A**) and mouse (**B**). Each cell in the plot marks the methylation score of 1 genomic region (rows) at each of the 20 cell types in human and 9 in mouse (columns). Up to 100 blocks with the highest methylation score are shown per cell type. The methylation score represents the number of fully unmethylated or methylated read pairs/total coverage for hypo- and hypermethylated blocks, respectively. (**C**) Schematic diagram depicting location of human cell-type-specific hypo- and hypermethylated blocks. Genomic annotations of cell-type-specific methylation blocks were determined by analysis using HOMER. (**D**) Distribution of human (left) and mouse (right) cell-type-specific methylation blocks relative to genomic regions used in the hybridization capture probes. Captured blocks with less than 5% variance across cell types represent blocks without cell-type specificity and were used as background. *P < 0.05 by χ^2^ test (degrees of freedom = 4). (**E**) Total number and top 5 TF binding sites enriched among cell-type-specific differentially methylated blocks (DMBs) in human (top) and mouse (bottom), using HOMER motif analysis (cumulative hypergeometric distribution statistic). As above, captured blocks with less than 5% variance across cell types were used as background.

Based on the above unsupervised clustering analysis, we selected a final set of reference methylomes used to identify differentially methylated cell-type-specific blocks. We excluded WGBS samples from bulk tissues and samples with low coverage. Subsets of some related cell types were considered together to form the final groups (i.e., monocytes grouped together with macrophages and colon grouped together with small intestine). We identified cell-type-specific differentially methylated blocks (DMBs) that contained a minimum of 3 CpG sites and overlapped with captured regions from our hybridization panels used in the analysis of cfDNA from serum. The co-methylation status of neighboring CpG sites in these blocks distinguished all cell types included in the final groups. Overall, we identified 2,876 human and 7,344 mouse highly cell-type-specific DMBs passing all thresholds (see Methods). The liquid biopsy study by Moss et al. (24) found that a subset of approximately 4000 informative CpG sites contribute to accurate deconvolution. In comparison, we utilized information from 28,810 CpG sites and 70,975 CpG sites within identified human and mouse cell-type-specific DMBs, respectively. In addition, the capture sequencing approach we took in this study allows for assessment of fragment-level methylation patterns as opposed to the single-site resolution of methylation arrays. The human and mouse DNA methylation blocks specific for these cell types can be found in Supplemental Tables 3, 4, and 15. A summary of human and mouse cell-type-specific methylation blocks is in Supplemental Table 5. Intriguingly, a variable number of blocks were identified for each cell type using the same thresholds. This is likely due to genuine biologic differences between cell types but also affected by the depth of coverage, purity, and degree of separation from other tissues and cell types currently included in the atlas. To visualize cell-type-specific DMBs, we created a methylation score that applies to both hypomethylated and hypermethylated DMBs. The score calculates the number of fully unmethylated read-pairs divided by total coverage for hypomethylated blocks (and vice versa for hypermethylated blocks). The heatmaps in Figure 3, A and B depict up to 100 blocks with the highest methylation score for each cell-type group.

### Differential DNA methylation is closely linked to the regulation of cell-type-specific functions.

We next sought to understand the role of cell-type-specific methylation in shaping cellular identity and function. For this, we identified genes adjacent to cell-type-specific methylation blocks and performed pathway analysis of annotated genes using both ingenuity pathway analysis (IPA) and genomic regions enrichment of annotations tool (GREAT) (33, 34). Important biological differences were observed in the gene sets identified based on specific processes unique to the cell types profiled. For example, the biological function of genes associated with immune cell–type-specific methylation reflects processes of leukocyte cell-cell adhesion, immune-response-regulating signaling, and hematopoietic system development (Supplemental Figure 4). In contrast, fatty acid metabolic process, lipid metabolism, and acute-phase response signaling were identified for hepatocytes. Significantly enriched biological pathways and functions for genes associated with differential methylation in each cell type examined are provided in Supplemental Table 6.

### Cell-type-specific DNA blocks are mostly hypomethylated and enriched at intragenic regions containing developmental TF binding motifs.

The majority of human and mouse cell-type-specific blocks identified here were hypomethylated, consistent with other studies (14, 17). In human samples we found 86% of cell-type-specific DMBs hypomethylated and only 14% hypermethylated. Strikingly, in the mouse samples, 98% of cell-type-specific DMBs were hypomethylated and only 2% were hypermethylated. The genomic locations of human and mouse cell-type-specific hypo- and hypermethylated blocks are depicted in the schematics in Figure 3C and Supplemental Figure 1B, respectively. Interestingly, regardless of directionality, the majority of cell-type-specific blocks were located within intragenic regions. To assess whether the genomic locations of cell-type-specific blocks show a distinct pattern, we compared their locations to the other captured blocks that do not vary among cell types (Figure 3D and Supplemental Table 7). We found that for both human and mouse, there was a significant enrichment of cell-type-specific blocks within intragenic regions relative to other captured regions (Fisher’s exact test, *P* < 0.05). There was also a significant relationship between directionality and intragenic distribution, with a significantly larger proportion of cell-type-specific blocks being hypermethylated in exons and hypomethylated in introns (χ^2^, degrees of freedom = 3, *P* < 0.05). The similar distribution of cell-type-specific methylation blocks in human and mouse suggests a conserved biological function of these genomic regions across species (Supplemental Figure 5).

**Figure 4:**
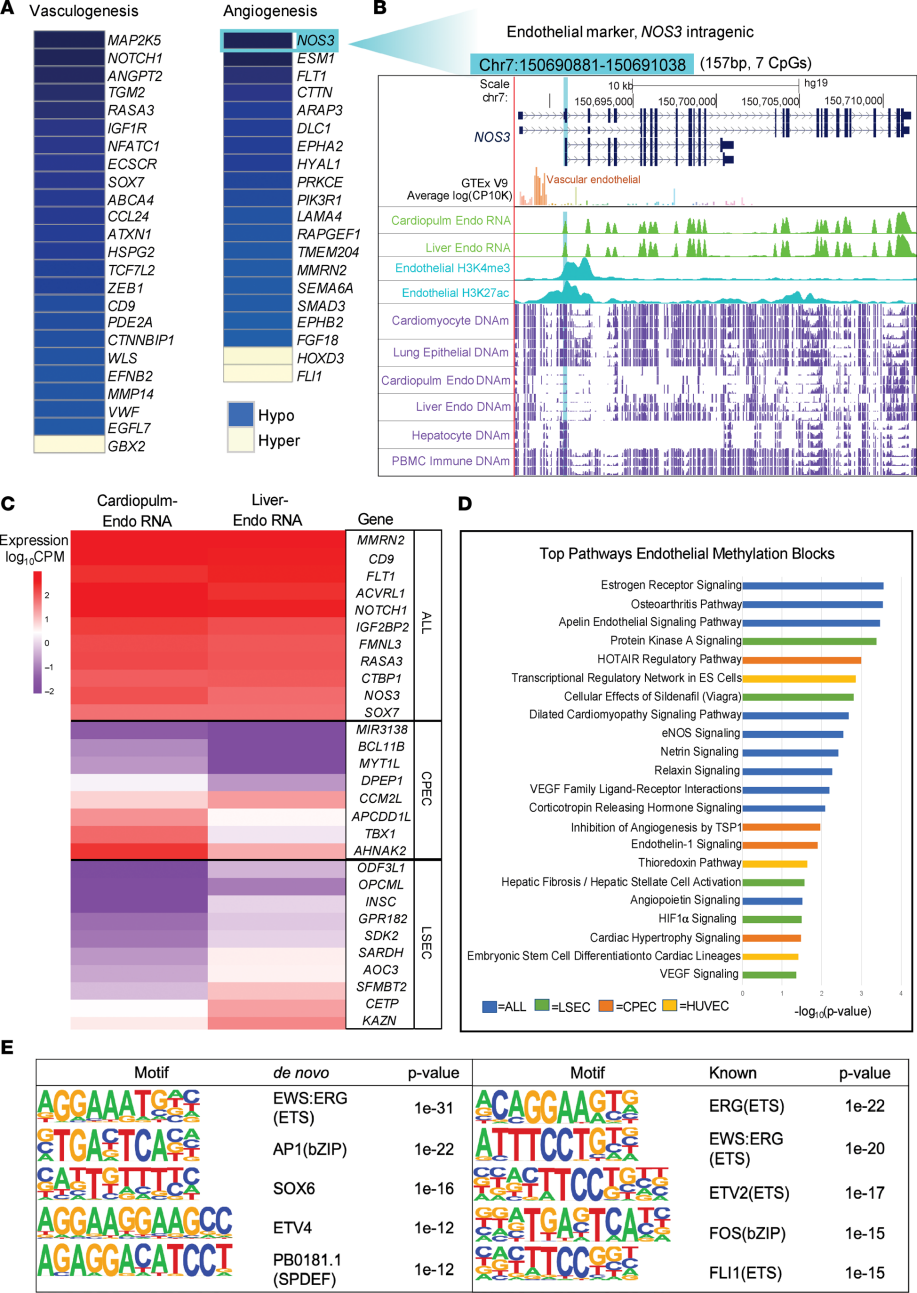
Methylation profiling of tissue-specific endothelial cell types reveals epigenetic heterogeneity associated with differential gene expression and biological functions. (**A**) Functions of genes adjacent to endothelium-specific methylation blocks (all *P* < 0.05). Blue color indicates nearby hypomethylated regulatory blocks. Yellow color indicates nearby hypermethylated regulatory blocks. (**B**) Example of the *NOS3* locus specifically unmethylated in endothelial cells. This endothelium-specific, differentially methylated block (DMB; highlighted in light blue) is 157 bp long (7 CpGs), and is located within the *NOS3* gene, an endothelium-specific gene (upregulated in paired RNA-seq data as well as in vascular endothelial cells, GTEx inset). The alignment from the UCSC genome browser (top) provides the genomic locus organization and is aligned with the average methylation (purple tracks) across cardiomyocyte, lung epithelial, liver sinusoidal endothelial cell (LSEC), cardiopulmonary endothelial cell (CPEC), hepatocyte, and immune (PBMC) samples (*n* = 3/cell-type group). Results from RNA-seq generated from paired cell types are depicted (green tracks) as well as peak intensity from H3K27ac and H3K4me3 published ChIP-seq data generated in endothelial cells (blue tracks). (**C**) Expression levels of genes adjacent to tissue-specific endothelial methylation blocks. Expression data were generated from paired RNA-seq of the same CPEC and LSEC populations used to generate methylation reference data. Pan-endothelial genes upregulated in both populations (ALL) are identified as common endothelium-specific methylation blocks to both LSEC and CPEC tissue–specific endothelial populations. (**D**) Pathways related to the biological function of genes containing endothelium-specific methylation blocks (all Benjamini-Hochberg–corrected *P* < 0.05 by right-tailed Fisher’s exact test). Unique pathways to tissue-specific endothelial cells are highlighted in distinct colors. (**E**) Top 5 transcription factor binding sites enriched among endothelium-specific hypomethylated blocks, using HOMER de novo and known motif analysis (cumulative hypergeometric distribution statistic). The background for the HOMER analysis consisted of 3,589 non–endothelial cell-type–specific hypomethylated blocks. HUVEC, human umbilical vein endothelial cell.

To further explore what common function these identified regions may have in human and mouse development, we performed motif analysis using HOMER to determine whether there were commonly enriched transcription factor (TF) binding sites (TFBSs) (35). MADS motifs bound by MEF2 transcription factors were significantly enriched in both human and mouse cell-type-specific hypomethylated blocks (Figure 3E). The MEF2 TFs are established developmental regulators with roles in the differentiation of many cell types from distinct lineages. In contrast, homeobox (HOX) motifs bound by several different HOX TFs were enriched in the human cell-type-specific hypermethylated blocks (Figure 3E). Specifically, HOXB13 was the top TF associated with binding at sites within the human hypermethylated DMBs. Recently, HOXB13 has been found to control cell state through binding to super-enhancer regions, suggesting a novel regulatory function for cell-type-specific hypermethylation (36). In addition to the common TFBSs enriched by all cell-type-specific blocks, endothelial cell–specific TFs were found to be enriched in the endothelial cell hypomethylated blocks, including EWS, ERG, FLI1, ETV2/4, and SOX6 (Figure 4E). Overall, these data indicate functions of these cell-type-specific blocks that represent cell-specific biology that is still underexplored.

### Methylation profiling of tissue-specific endothelial cell types reveals epigenetic heterogeneity associated with differential gene expression.

Endothelial damage has been implicated in the pathophysiology of radiation-induced cardiovascular disease, contributing to significant morbidity and mortality in patients with breast cancer (37–41). We thus generated tissue-specific endothelial methylomes paired with transcriptomes to be able to identify damage to distinct populations of microvascular and large vessel endothelial cell types, including coronary artery, pulmonary artery, cardiac microvascular, and liver sinusoidal endothelia. We also made use of publicly available liver sinusoidal endothelial (42) and umbilical vein endothelial methylomes (43) to complement our data (Supplemental Table 1). Previous studies support considering the vasculature of the heart and lung as an integrated system in the development of radiation damage due to the shared cardiopulmonary circulation (4). In addition, we were unable to identify methylation blocks that would separate cardiac from pulmonary microvascular endothelial cells in addition to LSECs and all other cell types in the human methylation atlas at the required specificity thresholds. Therefore, we merged cardiac and pulmonary endothelial cell types to generate a joint cardiopulmonary endothelial cell (CPEC) signal and identified the specific methylation blocks for CPEC, LSEC, and umbilical vein endothelial cell (HUVEC) types as distinct populations. We also identified pan-endothelial methylation blocks with methylation status in common to all endothelial cell populations relative to other cell types (Supplemental Figure 11, A–F). Pathway analysis of genes associated with these genomic regions confirmed endothelial cell identity based on genes involved in the regulation of vasculogenesis and angiogenesis (Figure 4A). In addition, unique pathways identified the tissue-specific epigenetic diversity of endothelial cell populations from different organs (Figure 4D). The DNA methylation status at several tissue-specific blocks was found to correspond to RNA expression levels of known endothelial cell–specific genes, confirming the identity of endothelial populations characterized (Figure 4, B and C, and Supplemental Table 8) (44–50). For example, hypomethylation was associated with increased expression at several pan-endothelial genes, including *NOTCH1*, *ACVRL1*, *FLT1*, *MMRN2*, *NOS3*, and *SOX7*. Likewise, hypomethylation at CPEC- or LSEC-specific genes was associated with differential expression when comparing the 2 populations to reflect tissue-specific differences.

### Methylated cfDNA changes indicate dose-dependent radiation damage in mice.

**Figure 5:**
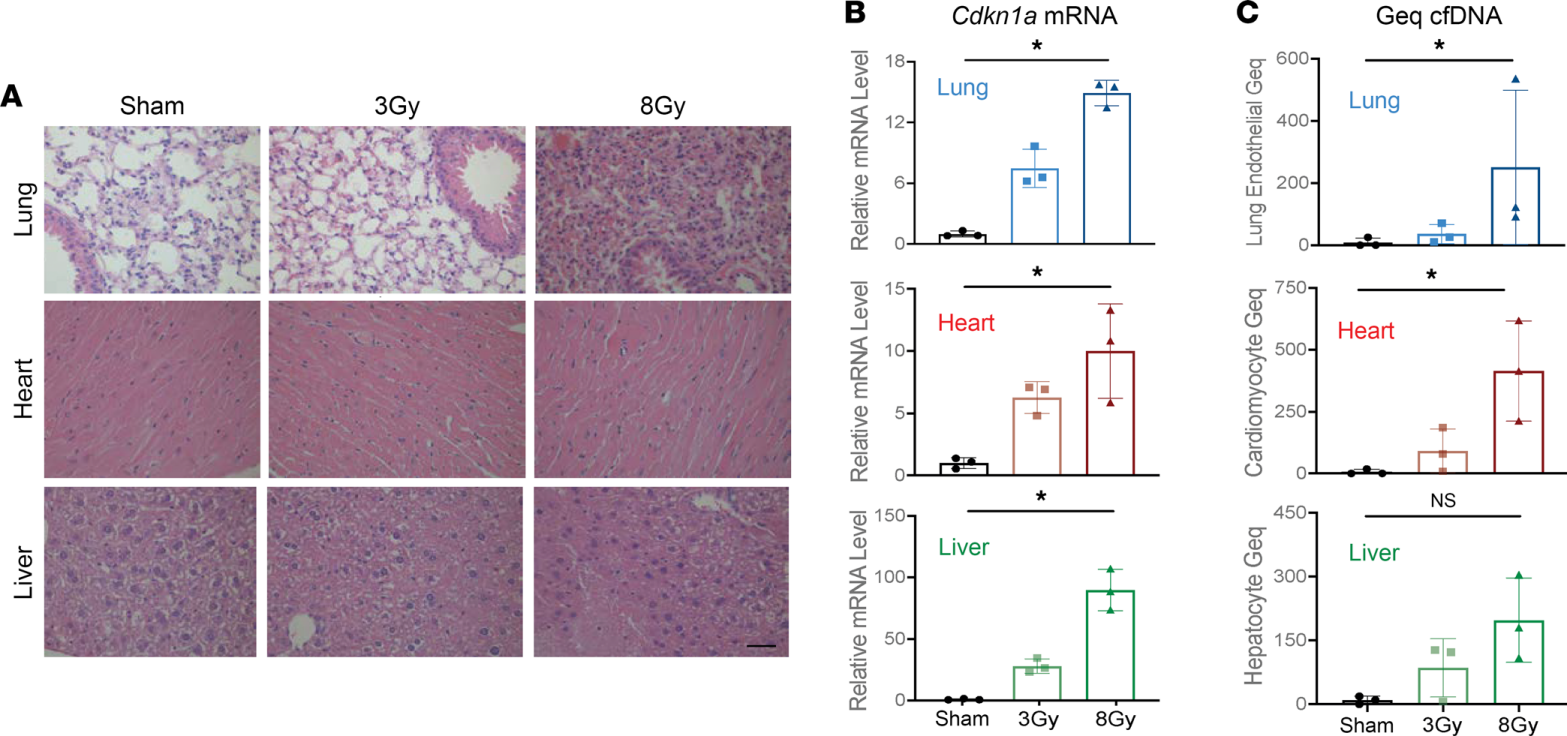
Dose-dependent radiation damage in mouse tissues correlates with the origins of methylated cfDNA in the circulation. (**A**) Representative H&E staining of lung, heart, and liver tissues from mice treated with 3 Gy or 8 Gy radiation compared to sham control. Scale bar: 200 μm. (**B**) qPCR analysis of *Cdkn1a* (p21) mRNA. The expression in each sample was normalized to *Actb* and is shown relative to the expression in the sham control. Mean ± SD; *n* = 3. Kruskal-Wallis test was used for comparisons among groups: lung tissue, *P* = 0.004; heart tissue, *P* = 0.025; liver tissue, *P* = 0.004. (**C**) Lung endothelial, cardiomyocyte, and hepatocyte methylated cfDNA in the circulation of mice treated with 3 Gy and 8 Gy radiation compared to sham control expressed in genome equivalents per mL serum (Geq/mL). cfDNA was extracted from 18 mice (*n* = 6 in each group), with cfDNA from 2 mice pooled in each methylome preparation. Mean ± SD; *n* = 3 independent methylome preparations. Kruskal-Wallis test was used for comparisons among groups. NS, *P* ≥ 0.05; *P < 0.05: lung endothelial, *P* = 0.01; cardiomyocyte, *P* = 0.01; hepatocyte, *P* = 0.13.

To explore the relationship between radiation-induced damage in tissues to changing proportions of cfDNA origins in the circulation, we used mice to model the response to exposure from different radiation doses. Mice received upper thorax radiation of 3 Gy or 8 Gy relative to sham control, forming 3 groups for comparison (Figure 1). Tissues and serum were harvested 24 hours after the last fraction of radiation and tissues in the path of the radiation beam (heart, lungs, and liver) were analyzed. H&E-stained sections showed a visible, dose-dependent impact of radiation on the tissues (Figure 5A). The changes were most apparent in tissue sections of the lungs, showing noticeable alveolar collapse with increased radiation dose. Liver tissues showed increased fibrosis with increased radiation doses and only minor changes were apparent in cardiac tissues matching with their lower sensitivity to radiation. Tissue effects were also assessed through qPCR analysis of established indicators of radiation effects, including expression of *Cdkn1a* (p21), that exhibited a dose-dependent increase in expression in response to radiation in all tissues (*P* < 0.05, Kruskal-Wallis test) (Figure 5B, Supplemental Figure 6, and Supplemental Table 9) (51).

To assess whether these damages to heart, lung, and liver are reflected by altered cfDNA composition in the circulation, we used capture sequencing of CpG-containing cfDNA fragments. For the data analysis, we applied the above-described cell-type-specific methylation blocks derived from the mouse methylation atlas to infer cfDNA origins. We found a significant dose-dependent increase in lung endothelial, cardiomyocyte, and combined solid organ cfDNA that correlated with radiation-induced cell death in the corresponding tissues (*P* < 0.05, Kruskal-Wallis test) (Figure 5C and Supplemental Figure 7G). The dose-dependent increase in hepatocyte cfDNA was not statistically significant and immune cell cfDNA showed no change between treatment groups (*P* ≥ 0.05, Kruskal-Wallis test) (Figure 5C and Supplemental Figure 7F). We conclude that changes in cfDNA fragments in the circulation can reveal the cellular source of radiation-induced damage in tissues.

### Radiation treatment of patients with breast cancer.

To evaluate whether changes in cfDNA patterns could indicate damage to tissues in patients treated with radiation, we collected serum samples from patients with breast cancer at 3 time points during their standard-of-care radiation therapy after surgery (Figure 1). A baseline sample was taken for each patient before onset of radiation therapy and a second end-of-treatment (EOT) sample was taken 30 minutes after the last treatment. Finally, a recovery sample was taken 1 month after completion of radiation therapy. Demographic information and clinical characteristics of patients enrolled in this study are provided in Supplemental Tables 10 and 11.

### Methylated cfDNA changes provide an estimate of tissue dose to indicate radiation-induced damage to healthy tissues.

**Figure 6:**
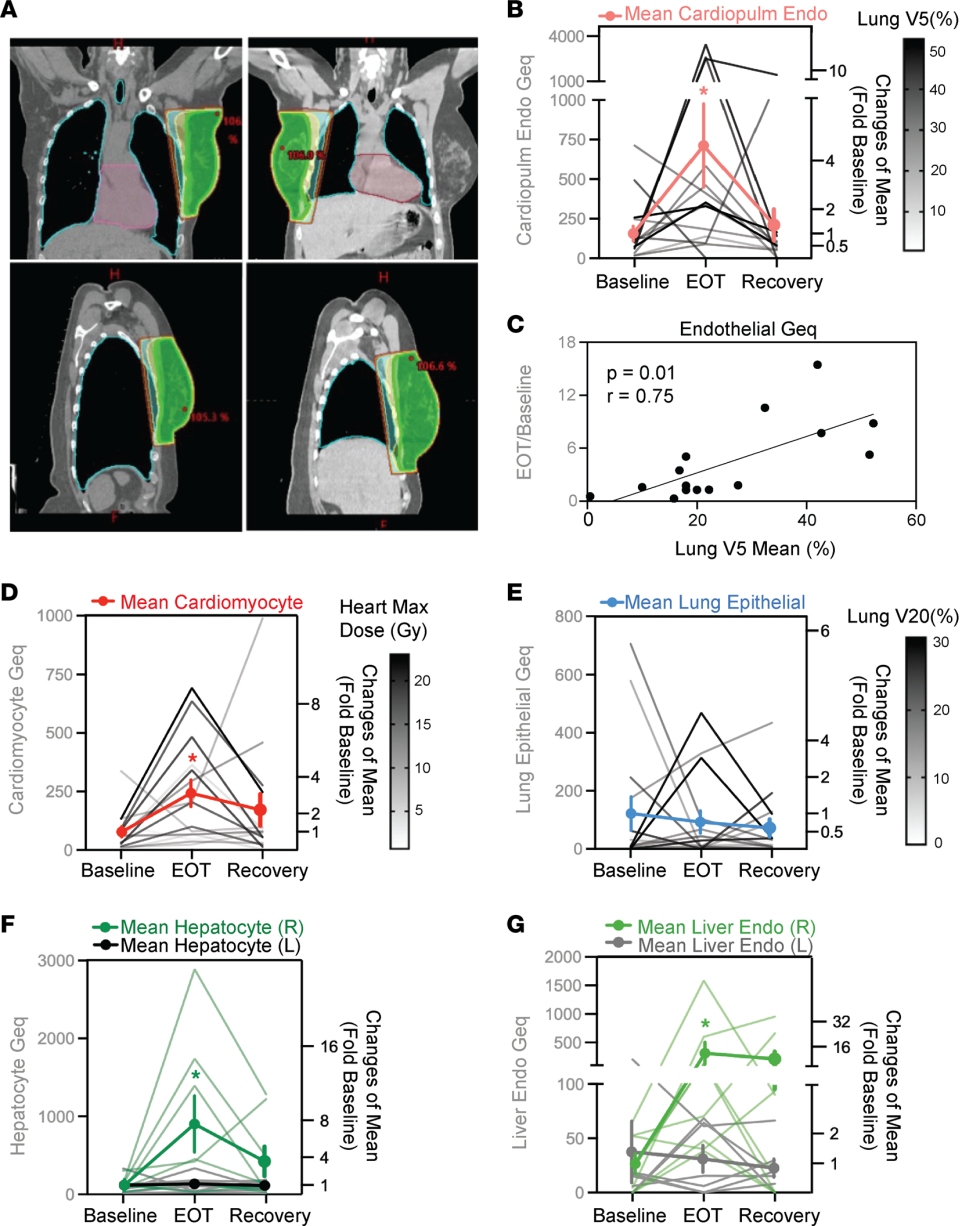
Methylated cell-type-specific cfDNAs provide an estimate of tissue dose to indicate radiation-induced damage to healthy tissues in patients with breast cancer. (**A**) Representative 3D-CRT treatment planning for patients with left-sided (left) and right-sided (right) breast cancer, respectively. The color map represents different radiation dose levels or isodose lines (green: 95% of prescription dose; isodose lines: yellow = 90%, cyan = 80%, orange = 70%, brown = 50%). (**B**, **D**, and **E**) Cardiopulmonary endothelial cell (CPEC), cardiomyocyte, and lung epithelial cfDNA (in Geq/mL) in serum samples. Fragment-level deconvolution used CPEC- (*n* = 99), cardiomyocyte- (*n* = 374), and lung epithelial cell–specific methylation blocks (*n* = 69), respectively. Friedman test compared paired results at baseline, end of treatment (EOT), and recovery time points. *P < 0.05; CPEC *P* = 0.03, cardiomyocyte *P* = 0.01, lung epithelial *P* = 0.99. Mean ± SEM fold change relative to baseline levels is shown in bold (*n* = 15). (**C**) Correlation of total endothelial cfDNA with dosimetry data. cfDNA is from deconvolution of pan-endothelial methylation blocks (*n* = 131), the mean volume of the lung receiving the 5-Gy dose is represented by Lung V5 Mean (%). Spearman’s correlation *r* was calculated, and considered significant when *P < 0.05. (**F** and **G**) Hepatocyte and liver sinusoidal endothelial cell (LSEC) cfDNA (in Geq/mL) in serum samples. Fragment-level deconvolution used hepatocyte (*n* = 200) and LSEC methylation blocks (*n* = 61). Mean ± SEM fold change relative to baseline levels is shown in bold (*n* = 8 right-sided, *n* = 7 left-sided breast cancer). Wilcoxon’s matched-pairs signed-rank test was used for comparison among groups. *P < 0.05; hepatocyte right-sided *P* = 0.02, hepatocyte left-sided *P* = 0.81, LSEC right-sided *P* = 0.02, and LSEC left-sided *P* = 0.93.

Due to close proximity with the target treatment area, the heart and lungs are common organs at risk for patients with breast cancer undergoing radiation therapy (Figure 6A). To assess therapy-induced lung damage, we examined patient serum for the presence of lung epithelial cfDNA. Interestingly, we did not find a significant increase in lung epithelial cfDNA across all patients (*P* ≥ 0.05, Friedman test) (Figure 6E). However, a few patients showed increased lung epithelial cfDNA, indicating lung damage that corresponded with increasing dose and volume of the ipsilateral lung receiving a 20-Gy dose (Lung V20). In addition to lung injury, cardiovascular disease is one of the most serious complications from radiation exposure that is associated with increased morbidity and mortality (6). Through deconvolution using cardiopulmonary endothelial (CPEC) and cardiomyocyte-specific DMBs, we found increased CPEC and cardiomyocyte cfDNA in serum, indicating significant cardiovascular cell damage across all breast cancer patients (*P* < 0.05, Friedman test) (Figure 6, B and D). Changes in total endothelial cfDNA after radiation correlated with the average volume of the lung receiving a 5-Gy dose (Lung V5 Mean) (Spearman’s *r* = 0.75, *P* < 0.05) (Figure 6C). In addition, changes in total endothelial cfDNA after radiation also correlated with the mean lung dose (Gy), total body mean dose (Gy), and the volume of the lung receiving a 20-Gy dose (Lung V20) (Supplemental Figure 8, D–F). Surprisingly, cardiomyocyte-specific methylated DNA in the circulation increased with the maximum radiation dose to the heart (Spearman’s *r* = 0.52, *P* = 0.05; Supplemental Figure 8A), but not the mean dose to the heart (Spearman’s *r* = 0.208, *P* ≥ 0.05) or volume of the heart receiving a 5-Gy dose (Heart V5) (Spearman’s *r* = 0.45, *P* ≥ 0.05). This suggests that radiation-induced damage of cardiomyocytes requires a threshold dose to cause cell death and matches with the relative resilience of this cell type compared with epithelial and endothelial cells.

### Radiation-induced hepatocyte and liver endothelial cfDNAs in patient with right- versus left-sided breast cancer.

While liver damage is not a common radiation-induced toxicity in patients with breast cancer, a substantial dose may still be administered to the liver, especially with right-sided tumors (Figure 6A). We used the top hepatocyte (*n* = 200) and liver sinusoidal endothelial (LSEC) DNA methylation blocks to assess the serum cfDNA sequence data for the presence of liver-derived DNA fragments. Surprisingly, in patients receiving radiation treatment of right-sided breast cancer, an increase in hepatocyte as well as LSEC methylated DNA in the circulation indicated significant radiation-induced cellular damage in the liver (*P* <0.05, Wilcoxon’s matched-pairs signed-rank test) (Figure 6, F and G). Elevated levels of either hepatocyte and/or LSEC cfDNA were detected in 7 of the 8 breast cancer patients with right-sided tumors. In contrast, there was no significant increase in hepatocyte or LSEC cfDNA in patients with left-sided breast cancer (*P* ≥ 0.05, Wilcoxon’s matched-pairs signed-rank test). The association of elevated liver-derived cfDNA with right-sided versus left-sided tumor location was also corroborated by correlation with the maximum dose administered to the liver (Gy) (Supplemental Figure 8, B and C).

### Detection of distinct endothelial and epithelial damage from radiation.

We observed distinct epithelial and endothelial cell–type responses to radiation across the different tissue cfDNAs profiled. Different responses to radiation were observed when comparing hepatocyte to lung epithelial damage (Figure 6E versus Figure 6F), demonstrating the ability of methylated cfDNA to distinguish between tissue-specific epithelial cell types from serum samples. Likewise, analysis for tissue-specific endothelial populations revealed differences in CPEC and LSEC responses to radiation (Figure 6B versus Figure 6G). In general, there was greater amount of damage in genome equivalents (Geqs) to the combined endothelium compared with the epithelium across different organs (Supplemental Figure 7E). Also, there was a 5-fold higher signal from CPEC cfDNA compared with lung epithelial cfDNA. The endothelium is formed by a layer of cells lining blood as well as lymphatic vessels, and turnover of this cell type may contribute to the high level of signal detected in the circulation (24). This could, however, also be a result of the different sensitivities of endothelial versus epithelial cell types to radiation-induced damage. Furthermore, in comparison with lung epithelial cell– and CPEC-derived cfDNA, sustained injury and delayed recovery is indicated by elevated cardiomyocyte cfDNA at the recovery time point (2-fold elevation from baseline) (Figure 6D). This may reflect important differences in cell turnover rates leading to differential processes of regeneration and repair in these cell types. Notably, 1 month after completion of radiation therapy, CPEC damage signatures detected from cfDNA had returned to baseline levels, whereas sustained higher cfDNAs from cardiomyocytes and liver cell types indicates lingering tissue remodeling. Taken as a whole, these findings demonstrate applicability of this approach to uncover distinct cellular damage in different tissues during the course of treatment by the analysis of blood samples.

## Discussion

This study demonstrates the feasibility of tissue-of-origin analysis of methylated cfDNA to monitor tissue responses to radiation exposure. The assignment of DNA fragments extracted from serum samples from patients undergoing treatment as well as from experimental animals to specific cell types required in-depth analysis of tissue- and cell-type DNA methylation patterns and generation of coherent atlases. We were positively surprised by the significant association of the cell-type-specific DNA methylation blocks with cell-type-specific gene expression, TF binding motifs, and signaling pathway regulation supporting the biological validity of the selected methylation blocks. We saw that cell-type-specific DNA methylation is well conserved across individuals from diverse donor populations that contributed to the methylation atlas. This suggests broad applicability in the monitoring of tissue damage. Also, this implies that disease- or age-related changes in DNA methylation occur outside the cell-type-specific blocks and thus will exert their impact without altering the features defining a particular cell type.

To enhance the quality and sensitivity of the analyses, we developed human and mouse reference methylation atlases that were tailored to this study. Low-integrity cfDNAs were isolated from human and mouse serum samples with an optimized capture-sequencing methodology that increased sequencing coverage. This improves the sensitivity of deconvolution when applying our sequencing-based, fragment-level probabilistic model. These improvements allow for accurate cellular assignment of cfDNA fragments present in serum. We made an effort to include major cell types within target organs at risk as well as those thought to contribute to cfDNA in the circulation of healthy individuals (5, 6, 24). While our reference atlas is limited to cell types that could be purified sufficiently for methylome analysis, this reference-based approach allows for direct inference of the relationship between changing cellular composition of cfDNA and response to radiation treatment over time. When evaluating our approach, we also directly compared cfDNAs extracted from serum and plasma samples harvested from the same donor. The results were highly correlated when comparing methylation status and cellular origins of cfDNA (Supplemental Figure 13). Interestingly, we found slightly less variation across donors in the cell-type proportions contributing to the cfDNA extracted from serum compared to plasma (details in the Supplemental Methods section; Supplemental Figure 13).

Comparing the origins of elevated cfDNA after upper thorax exposure to radiation showed similar changes in both human and mouse serum samples, providing further validation of the approach. In both human and mouse, there was a significant increase in endothelial cell and cardiomyocyte cfDNA in the circulation after radiation. Likewise, there was an overall increase in cfDNA derived from any solid-organ tissue after radiation (Supplemental Figure 7, B and G). We also detected treatment-induced increases in breast basal and luminal epithelial cfDNA across all patients with breast cancer (Supplemental Figure 7, C and D) and an increase in mammary epithelial cfDNA in mice exposed to radiation (Supplemental Figure 7H). The total cfDNA concentration was not increased by radiation exposure of mice, as also reported by others (52), although some patients with breast cancer showed an increase in total cfDNA at the end of the treatment cycle (Supplemental Tables 12 and 13). Although we saw parallels in the cellular impact of radiation, the genomic alignment of the human and mouse atlases is limited by the current scarcity of deep-sequencing WGBS data from purified murine cell types and low cross-species mapability of epigenetic regions when converting coordinates between the genomes (Supplemental Table 14) (53, 54).

The vascular endothelium is among the cell types known to be affected by radiation exposure, although the role of radiation-induced endothelial damage in mediating acute and chronic adverse effects has yet to be fully understood (4, 41). Recent studies suggest that compromised endothelial cell function also impairs wound healing by depriving tissues of signals necessary for regeneration and contributing to accelerated aging of the hematopoietic and vascular systems (55–57). From an initial comparison we did see a greater absolute increase in signal measured in Geq from the endothelium compared with epithelium in different organs. This may indeed reflect the expected higher sensitivities of endothelial cells to radiation, although the endothelial location bordering the blood stream may impact the amplitude of the signal detected from cfDNA as well. We also established distinct methylation patterns that reflect organ-specific differences between endothelial cell populations in different tissues. For the present study, we generated tissue-specific endothelial methylomes to assess potentially distinct sensitivities of CPECs and LSECs to radiation. While all endothelial cells share methylation patterns that are common to their lineage (Figure 2, A and B), we were pleasantly surprised by the diverse injury patterns observed when juxtaposing CPEC to LSEC signals that reflect distinct liver exposure based on the sidedness of the cancer location. Interestingly, 1 month after completion of radiation therapy the majority of damage signatures detected in serum had returned to baseline levels. However, ongoing tissue repair and remodeling at this recovery time point is indicated by sustained elevation of cardiomyocyte cfDNA as well as hepatocyte- and liver sinusoidal endothelia–derived cfDNAs in patients with right-sided breast cancers (Figure 6). Exploration of these tissue- and cell-type-specific differences and their distinct time courses may shed light on previously unknown mechanisms of radiation-induced damage and repair.

The liver is not a common organ at risk for radiation-induced toxicity in breast cancer. Thus, we were surprised to find an increase in hepatocyte and LSEC methylated DNA in the circulation of patients receiving radiation treatment for right-sided breast cancer. Previous studies have not found a relationship between breast cancer radiation and overt liver fibrosis, even at doses higher than 40 Gy, although increased hepatic exposure is expected in radiation treatment of patients with right-sided breast cancer (58). The liver is an interesting organ in this respect, and many factors, including radiation dose, fraction size, volume of the liver radiated, and preexisting hepatic function may account for the discrepancy in the known association between exposure and clinical presentation (59, 60). The elevated liver cfDNAs we detected demonstrate the sensitivity of the approach to identify previously unknown cell types and tissues affected by radiation treatment. Despite being at subclinical levels, this may become relevant in patients with hepatotoxic therapy regimens or comorbidities.

Only 3 patients (RT-102, RT-103, and RT-107) presented with grade 2 skin toxicity based on Common Terminology Criteria for Adverse Events (CTCAE v5) within our study timeline up to 1 month after completion of radiation therapy. While this is a small number, we detected significantly increased breast basal and luminal epithelial damage in these 3 patients that corresponded with the clinical presentation (Supplemental Figure 7, C and D). We also detected elevated breast epithelial injury in patients who underwent mastectomy, were treated with proton beam therapy (PBT), and had a higher overall dose administered — all clinical factors associated with elevated risk of skin toxicity. Although the findings from this study are highly encouraging, additional studies with a larger sample size are needed to further define the relationship between tissue dose administered and cell-type-specific methylated cfDNA changes in the circulation. As a future direction, it will be interesting to explore whether these methylated cfDNA changes occurring early on after radiation treatment can predict later onset of toxicity. We hypothesize that circulating cfDNA analyses may be used to assess tissue toxicity profiles independent of the presentation of clinical symptoms and compare tissue effects of different radiation treatment approaches such as 3-dimensional conformal RT (3D-CRT), PBT, and intensity-modulated radiation therapy. Likewise, exploration of regional variation in tissue-specific responses to radiation may offer opportunities to reduce tissue toxicity (61).

In conclusion, as a proof of concept we show that cell-type-specific methylation signatures can be applied to detect cellular injury from radiation treatment using DNA shed into the circulation. In mice, paired tissue and serum analysis allowed for a direct comparison of tissue damage and its cfDNA correlates in serum samples. Treatment planning for patients with breast cancer provided an estimate of the organ volume affected and radiation dose for organs at risk, including the heart, lungs, and liver. We found a striking degree of correlation between the planned tissue radiation dose administered and the observed degree of distinct cellular damage indicated by changes in cell-type-specific methylated cfDNA. We conclude that the minimally invasive detection of methylated cfDNA from serum samples can indicate organ-specific damage, reveal biologically effective exposure, and identify cell types affected by radiation treatment.

## Methods

### Human breast cancer patient serum sample collection.

Serial serum samples were collected from 15 patients with breast cancer at baseline (before radiation treatment), EOT (30 minutes after the last radiation treatment), and recovery (1 month after cessation of radiation treatment), thus allowing for a within-patient internal control and baseline. A schematic of the time series for sample collection can be found in Figure 1. For serum isolation, peripheral blood (~8–12 mL) was collected in red-top venous puncture tubes and allowed to clot at room temperature for 30 minutes before centrifugation at 1500*g* for 20 minutes at 4°C to separate the serum fraction. Patients received either 3D-CRT or a combination of PBT and 3D-CRT. Patient characteristics and treatment details including radiation dosimetry are summarized in Supplemental Tables 10 and 11.

### Mouse serum and tissue collection.

C57BL/6 mice (*n* = 18) from The Jackson Laboratory were x-ray irradiated (~1.67 Gy/min; X-Rad 320, Precision X-Ray Inc.; filter, 0.75 mm tin/0.25 mm copper/1.5 mm aluminum, 320 kV, 12.5 mA) to the upper thorax at different doses (sham control, 3 Gy, 8 Gy) for 3 consecutive treatments. The irradiated lung and heart tissues received either 3 × 3 Gy, or 3 × 8 Gy of radiation. The liver was partially irradiated, depending on the layout of the liver in the irradiated area. We estimate that around 40%–60% of the liver received either 3 × 3 Gy or 3 × 8 Gy of radiation based on the assumption that the dose is identical to the tissue exposed as the unit of energy per unit mass of tissue (1 Gy = 1 Joule/kilogram). Serum and tissues were collected 24 hours after the last radiation dose. For serum isolation, blood was collected via cardiac puncture (~1 mL) and allowed to clot at room temperature for 30 minutes before centrifugation at 1500*g* for 20 minutes at 4°C to separate the serum fraction. Heart, lung, and liver tissues were harvested and sectioned to be both flash frozen and formalin fixed for subsequent analysis.

### Cell isolation to generate reference methylomes.

Reference methylomes were generated for mouse immune cell types and human endothelial cell types to complement publicly available data sets. Peripheral blood and bone marrow were isolated and spleens from healthy C57BL/6 mice were dissociated to single cells and FACS isolated using cell-type-specific antibodies. Buffy coat (*n* = 4), bone marrow (*n* = 3), CD19^+^ B cell (*n* = 1), CD4^+^ T cell (*n* = 1), CD8^+^ T cell (*n* = 1), and Gr1^+^ neutrophil (*n* = 1) methylomes were generated after cell isolation using the following antibodies: FITC anti–mouse CD45 (catalog 103107), Alexa Fluor 647 anti–mouse CD3 (catalog 100209), Brilliant Violet 711 anti–mouse CD4 (catalog 100549), Brilliant Violet 421 anti–mouse CD8a (catalog 100737), PE anti–mouse CD19 (catalog 152407), and PE/Cy7 anti–mouse Ly-6G/Ly-6C (Gr-1) (catalog 108415) (all BioLegend; diluted 1:20). Cryopreserved passage 1 human LSECs were purchased from ScienCell research laboratories (catalog 5000). Cryopreserved passage 2 human coronary artery endothelial cells (catalog C-14022), cardiac microvascular cells (catalog C-14029), and pulmonary artery endothelial cells (catalog C-14024) were isolated from single donor healthy human tissues purchased from PromoCell. Paired RNA-seq data were generated from the same cell populations used for DNA methylation profiling to validate the identity of purchased cell populations through analysis of cell-type expression markers.

### Isolation of circulating cfDNA.

Circulating cfDNA was extracted from 3 to 4 mL of human serum or plasma or 0.5 mL of mouse serum, using the QIAamp Circulating Nucleic Acid kit (Qiagen) according to the manufacturer’s instructions. cfDNA was quantified via Qubit fluorometer using the dsDNA High Sensitivity Assay Kit (Thermo Fisher Scientific). Additional size selection using Beckman Coulter beads was applied to remove high-molecular-weight DNA reflective of cell lysis and leukocyte contamination, as previously described (62). The same bead-based size selection was applied to all samples that were acquired through standardized serum isolation and cfDNA extraction protocols. This DNA purification step has been demonstrated to remove contaminating traces of high-molecular-weight genomic DNA (~10 kb) from cfDNA released into the bloodstream through a natural process of cell death (~150–200 bp). This method also served to concentrate the samples to the desired input volume before bisulfite conversion. Fragment size distribution of isolated cfDNA after size selection was validated on the 2100 Bioanalyzer TapeStation (Agilent Technologies) (Supplemental Figure 13 and Supplemental Methods).

### Isolation and fragmentation of genomic DNA.

Genomic DNA from tissues was extracted with the DNeasy Blood and Tissue Kit (Qiagen) following the manufacturer’s instructions and quantified via the Qubit fluorometer dsDNA BR Assay Kit (Thermo Fisher Scientific). Genomic DNA was fragmented via sonication using a Covaris M220 instrument to the recommended 150–200 bp before library preparation. Lambda phage DNA (Promega Corporation) was also fragmented and included as a spike-in to all DNA samples at 0.5% (w/w), serving as an internal unmethylated control. Bisulfite conversion efficiency was calculated through assessing the number of unconverted C’s on unmethylated lambda phage DNA. The SeqCap Epi capture pool contains probes to capture the lambda genomic region from base 4500 to 6500.

### Bisulfite capture-sequencing library preparation.

Bisulfite capture-sequencing libraries were generated from either cfDNA or fragmented genomic DNA using the same protocol. As a first step, WGBS libraries were generated using the Pico Methyl-Seq Library Prep Kit (Zymo Research) with the following modifications: Bisulfite conversion was carried out using the EZ DNA Methylation Gold kit (Zymo Research) instead of the EZ DNA Methylation-Lightning Kit. For mouse samples, cfDNA from 2 mice in the same group was pooled as the input of library preparation. An additional 2 PCR cycles were added to the recommended cycle number based on the total amounts of input cfDNA. WGBS libraries were eluted in 15 μL of 10 mM Tris-HCl buffer, pH 8. Library quality control was performed with an Agilent 2100 Bioanalyzer and quantity determined via the KAPA Library Quantification Kit (KAPA Biosystems).

Cell-free WGBS libraries were pooled to meet the required 1 μg DNA input necessary for targeted enrichment. However, no more than 4 WGBS libraries were pooled in a single hybridization reaction and the 1 μg input DNA was divided evenly between the libraries to be multiplexed. Hybridization capture was carried out according to the SeqCap Epi Enrichment System protocol (Roche NimbleGen, Inc.) using SeqCap Epi CpGiant probe pools for human samples and SeqCap Epi Developer probes for mouse samples with xGen Universal Blocker-TS Mix (Integrated DNA Technologies) as the blocking reagent. Washing and recovering of the captured library, as well as PCR amplification and final purification, were carried out as recommended by the manufacturer. The capture library products were assessed by Agilent Bioanalyzer DNA 1000 assays (Agilent Technologies, Inc.). Bisulfite capture-sequencing libraries with inclusion of 15%–20% spike-in PhiX Control v3 library (Illumina) were clustered on an Illumina Novaseq 6000 S4 flow cell followed by 150-bp paired-end sequencing. The individual sample-to-sample variability of sequencing parameters was minimal and the coverage was highly correlated amongst the sequencing libraries (Supplemental Figure 14).

### Bisulfite sequencing data alignment and preprocessing.

Paired-end FASTQ files were trimmed using TrimGalore (v0.6.6) with parameters “--paired -q 20 --clip_R1 10 --clip_R2 10 --three_prime_clip_R1 10 --three_prime_clip_R2 10” (63). Trimmed paired-end FASTQ reads were mapped to the human genome (GRCh37/hg19 build) using Bismark (v0.22.3) (64) with parameters “--non-directional”, and then converted to BAM files using Samtools (v1.12) (65). BAM files were sorted and indexed using Samtools (v1.12). Reads were stripped from non-CpG nucleotides and converted to BETA and PAT files using wgbstools (v0.1.0), a tool suite for working with WGBS data while preserving read-specific intrinsic dependencies (22, 66).

### Reference DNA methylation data from healthy tissues and cells.

Controlled access to reference WGBS data from normal human tissues and cell types was requested from public consortia participating in the International Human Epigenome Consortium (IHEC) (67) and upon approval downloaded from the European Genome-Phenome Archive (EGA), Japanese Genotype-phenotype Archive (JGA), database of Genotypes and Phenotypes (dbGAP), and ENCODE portal data repositories. Reference mouse WGBS data from normal tissues and cells were downloaded from selected GEO and SRA data sets. Additional information and citation of reference methylation data used in this study can be found in Supplemental Methods and Supplemental Tables 1 and 2.

### Segmentation and clustering analysis.

We segmented the genome into blocks of homogeneous methylation, as described by Loyfer et al., using wgbstools (with parameters segment --max_bp 5000) (22, 66). In brief, a multichannel dynamic programming segmentation algorithm was used to divide the genome into continuous genomic regions (blocks) showing homogeneous methylation levels across multiple CpGs for each sample. We applied the segmentation algorithm to 297 human reference WGBS methylomes and retained 351,395 blocks covered by the hybridization capture panel used in the analysis of cfDNA. Likewise, segmentation of 109 mouse WGBS data sets from healthy cell types and tissues identified 1,344,889 blocks covered by the mouse hybridization capture panel. The probed regions span 80 Mb (~20% of CpGs) on the human panel and 75 Mb (~50% of CpGs) on the mouse panel. Relative to the Infinium MethylationEPIC BeadChip, a commonly used human microarray (27), the human capture panel provides a 7-fold increase in CpG sites profiled. Likewise, the mouse capture panel provides a 35-fold increase in CpG sites profiled relative to the Infinium Mouse Methylation BeadChip. The human blocks had a median length of 326 bp (interquartile range [IQR] = 890 bp) and 8 CpGs (IQR = 14 CpGs). Similarly, the mouse blocks had a median length of 770 bp (IQR = 1,252 bp) and 7 CpGs (IQR = 7 CpGs). The hierarchical relationship between reference tissue and cell-type WGBS data sets was visualized as a tree dendrogram. The top 30,000 most variable methylation blocks containing at least 3 CpG sites and coverage across 90% of samples were selected, irrespective of sample cell-type group. We computed the average methylation for each block and sample using wgbstools (--beta_to_table). Trees were assembled using the unweighted pair-group method with arithmetic mean (68), using scipy (v1.7.1) (69) and L1 distance, and then visualized in R with the ggtree package (v2.4.1) (70). The similarity between samples was assessed by the degree of variation in distance between samples of the same cell type (average 23,056) compared to samples between different cell types (average 273,018). Dimensional reduction was also performed on the selected blocks using the UMAP package (v0.2.8.2.0). Default UMAP parameters were used (15 neighbors, 2 components, Euclidean metric, and a minimum distance of 0.1).

### Identification of cell-type-specific methylation blocks.

Tissue- and cell-type-specific methylation blocks were identified from reference WGBS data using custom scripts (Supplemental Code and Supplemental Methods). We performed a one-versus-all comparison to identify DMBs unique for each group. This was done separately for human and mouse. From this we first identified blocks covering a minimum of 3 CpG sites, with lengths of less than 2 kb and at least 10 observations. Then, we calculated the average methylation per block/sample, as the ratio of methylated CpG observations across all sequenced reads from that block. Differential blocks were sorted by the margin of separation, termed “delta beta,” defined as the minimal difference between the average methylation in any sample from the target group versus all other samples. Then, we computed the “soft margin” between target samples and background samples, allowing for some outliers using percentiles (Supplemental Figure 15). For all hypomethylated markers we calculated the difference between the 80th percentile of the methylation status in the target group (--target.quant 0.2) and the 10th percentile of the methylation status in the background group (--bg.quant 0.1). We selected blocks with a soft margin of 0.4 or greater for human and 0.35 or greater for mouse. Blocks with a (–) direction are hypomethylated and (+) direction are hypermethylated, defined as a direction of methylation in the target cell type relative to all other tissues and cell types included in the atlas. Additional separation of endothelial cell populations from different tissues was performed to identify unique markers for liver endothelial versus cardiopulmonary endothelial blocks that did not overlap. Separately, pan-endothelial blocks were identified with methylation status in common to all endothelial cell populations. Similarly, individual immune cell-type-specific methylation blocks were identified for purified cell populations. In addition, bulk immune blocks were identified with methylation status in common to all hematopoietic cell populations. The bulk immune methylation blocks were found to separate all hematopoietic cell types from solid organ cell types of different lineages and were used for deconvolution in the circulation. The solid organ compartment was then further parsed into individual cell-type contributors as specified in Column F titled “Atlas Groups” in Supplemental Tables 1 and 2. For some cell types, a reduced subset of blocks (i.e., top 200) were used for deconvolution in the circulation if the original number identified was greater than 1 SD above the mean. Selected human and mouse blocks for cell types of interest that were used to identify origins of cfDNA can be found in Supplemental Tables 3 and 4. Extended cell-type-specific blocks for purified populations of endothelial and immune cell types can be found in Supplemental Tables 8 and 15.

### Likelihood-based probabilistic model for fragment-level deconvolution.

The cell-type origins of cfDNA were determined using a probabilistic fragment-level deconvolution algorithm. Using this model, the likelihood of each cfDNA molecule was calculated using a fourth-order Markov model, by which methylation of each CpG site directly depends on up to 4 adjacent previous sites within each fragment. We estimated these parameters for each differential block at every tissue and cell-type, and then used Bayes’ theorem to infer the posterior probability of cell of origin for each fragment, based on its complete methylation pattern. The model was trained on reference bisulfite sequencing data from normal cells and tissues of known identity to learn the distribution of each marker in the target tissue/cell population of interest compared to background. Then the model was applied to predict the origins of each cfDNA molecule. The joint probability of each cfDNA molecule (methylation patterns and cellular origin) is calculated based on the likelihood of the methylation pattern (using the parameters for that cell type) times the prior probability that a read is originating from the target cell type. A prior probability of 0.1 was used for the combined endothelial cell type group and 0.85 for the combined immune cell type group, as expected based on findings in previous reports (24). A prior probability of 0.05 was used for all other solid organ cell types. Finally, each fragment is assigned to the cell type of origin with the maximal posterior probability (“hard” assignment). The proportion of molecules (fragments) assigned to the tissue of interest across all cell-type-specific markers was then averaged and used to determine the relative abundance of cfDNA derived from that tissue in each respective sample. We then adjusted the resulting proportions from all cell types to have a sum of 1 by imposing a normalization constraint. Tissue-specific endothelial cell types were normalized within the predicted total endothelial proportion identified by pan-endothelial markers in common to all endothelial cell types. Predicted cell type proportions were converted to genome equivalents and are reported as Geq/mL through multiplying the relative fraction of cell-type-specific cfDNA times the concentration of cfDNA (ng/mL) by the mass of the human haploid genome (3.3 × 10^–12^ g) or the mouse haploid genome equivalent (3.0 × 10^–12^ g).

### In silico simulations and WGBS deconvolution.

In silico mix-in simulations were performed using wgbstools (v0.1.0) (66) to validate the fragment-level deconvolution algorithm at the identified cell-type-specific blocks (Supplemental Figures 9–12), as previously described (22, 24). For each cell type profiled, we mixed known proportions of target fragments into a background of leukocyte fragments using wgbstools mix_pat. The leukocyte fragments were obtained from *n* = 4 buffy coat samples in mouse and *n* = 5 buffy coat samples in human. We performed 3 replicates for each admixture ratio assessed (0.05%, 0.1%, 0.5%, 1%, 2%, 5%, 10%, 15%), which were analyzed as described above, and present the average predicted proportion and standard deviation across all replicates. Model accuracy was assessed through correct classification of the actual percentage target mixed.

### Functional annotation and pathway analysis.

Cell-type-specific methylation blocks were annotated and motif analysis was performed using HOMER (v4.11.1) (35) and the annotatePeaks.pl and findMotifsGenome.pl functions. The top 5 motifs based on *P* value were selected from each analysis. Pathway analysis of genes adjacent to identified tissue and cell-type-specific methylation blocks was performed using IPA (34) (Qiagen) and GREAT (33). GeneSetCluster was used to cluster identified gene set pathways based on shared genes (71). Cross-species comparison of identified human and mouse cell-type-specific methylation blocks was performed using the UCSC Genome Browser LiftOver tool and the hg19ToMm9.over.chain.gz file (Supplemental Table 14).

### Genome browser visualization.

Endothelial methylomes and paired transcriptomes were uploaded as custom tracks for visualization on the UCSC genome browser (72). Methylomes were converted to bigwig format using the wgbstools beta2bw. Enrichment for chromatin marks was assessed through analysis of published H3K27ac and H3K4me3 ChIP-seq data (46). GTEx single-nucleus RNA-seq data were acquired from the GTEx v9 portal (gtexportal.org) (73) and analyzed using R (v4.1.3). Counts per ten thousand reads (CP10K) of *NOS3* were log-transformed and averaged for each specific cell type. Color represents the general cell type and intensity of color represents the number of cells expressing *NOS3*.

### Statistics.

Statistical analyses for group comparisons and correlations were performed using Prism (GraphPad Software) and R (v4.1.3). A correlation analysis was performed to assess relationship between changing cfDNAs and dose using Spearman’s rank correlation coefficient. Statistically significant comparisons are shown, with significance defined as *P* less than 0.05. Correction for multiple hypothesis comparisons was performed using the Benjamini-Hochberg method–corrected *P* value to control the false discovery rate (FDR) from multiple pathways being tested against each gene set. A 2-stage linear step-up procedure of Benjamini, Krieger, and Yekutieli was performed for *P* value adjustment from multiple outcome measures.

### Study approval.

Patients with breast cancer undergoing adjuvant radiation therapy were enrolled and provided signed informed consent in this IRB-approved study at Medstar Georgetown University Hospital (IRB protocol 2013-0049). Animal studies were approved by the Georgetown University IACUC (protocol 2017-0029).

### Data availability.

Raw sequencing files for DNA methylation data have been deposited in the database of Genotypes and Phenotypes (dbGAP) under study accession no. phs003290.v1.p1. Additional DNA methylation data for human and mouse samples are available in the NCBI Gene Expression Omnibus (GEO) under study accession no. GSE200187 (in BETA and PAT file formats). The BETA files (a wgbstools-compatible binary format) contain position and average methylation information for single CpG sites. The PAT files contain fragment-level information (including CpG starting index, methylation pattern of all covered CpGs, and number of fragments with exact multi-CpG pattern). Raw and normalized RNA-seq data have been deposited in GEO under study accession no. GSE200095. Availability of previously published and publicly available WGBS data from healthy cell types and tissues used in this paper are described in Supplemental Table 1 for human and Supplemental Table 2 for mouse. Code is available in Supplemental Code as well as at github.com/nloyfer/wgbs_tools and github.com/nloyfer/MarkovDeconv. Any additional information required to reanalyze the data reported in this paper is available from corresponding author AW upon request.

## Author contributions

MEM, AJK, SMR, EB, KU, and SR facilitated collection and processing of enrolled breast cancer patient samples. EB performed chart review and organized breast cancer patient data. MEM, AJK, and SMR performed bisulfite-capture sequencing library preparation. MEM and AJK purified cell types and performed reference methylation data generation and validation. YTL and HHL facilitated radiation treatment of mice. MEM and MOS facilitated serum and tissue collection from mice. AJK performed RT-qPCR and RNA-seq analyses. NS performed histological analysis of mouse tissues. MEM and NL performed bioinformatics analysis of WGBS data. MEM processed data for identification of mouse and human cell-type-specific differential methylation. NL and TK created wgbstools for bioinformatics read-specific processing of WGBS data. NL and TK generated all scripts for WGBS data processing, segmentation, and identification of cell-type-specific methylation blocks. NL, SSP, and TK developed the fragment-level deconvolution model for cfDNA analysis. SSJ, APM, and ADM advised on statistical and computational data analysis. MEM and AW wrote the manuscript and generated all figures. AW, ATR, and KU conceived the study design and provided interpretation of results. Relative overall contribution was used as the method to assign the authorship order. All authors critically reviewed and approved the manuscript.

## Supplementary Material

Supplemental data

Supplemental tables 1-15

Supporting data values

## Version 1 06/15/2023

In-Press Preview

## Version 2 07/24/2023

Electronic publication
