# Supplementary material for: Circulating cell-free methylated DNA reveals tissue-specific, cellular damage from radiation treatment
Source: JCI Insight. 2023 Jul 24;8(14):e156529. doi: 10.1172/jci.insight.156529 (PMC10443812; doi:10.1172/jci.insight.156529)
Supplement: Supplemental data [file jciinsight-8-156529-s081.pdf]

# Supplemental Information

Supplement to:

“Circulating cell-free methylated DNA reveals tissue-specific cellular damage from radiation treatment.” McNamara *et al.* 2023.

**Contents:**

Supplemental Materials and Methods

Supplemental References

Supplemental Figures and Legends

Legends for Supplemental Tables

## Supplemental Materials and Methods

*Processing of human serum and plasma samples*

Circulating cell-free DNA was extracted from 3 to 4 mL human serum or plasma or 0.5 mL mouse serum, using the QIAamp Circulating Nucleic Acid kit (Qiagen) according to the manufacturer's instructions. Cell-free DNA was quantified via Qubit fluorometer using the dsDNA High Sensitivity Assay Kit (Thermo Fisher Scientific). Additional size selection using Beckman Coulter beads was applied to remove high-molecular weight DNA reflective of cell-lysis and leukocyte contamination as previously described (1). The same bead-based size selection was applied to all samples that were acquired through standardized serum isolation and cfDNA extraction protocols. This method also served to concentrate the samples to the desired input volume before bisulfite conversion. Fragment size distribution of isolated cfDNA was validated on the 2100 Bioanalyzer TapeStation (Agilent Technologies).

Control human serum and plasma from healthy adult donors was purchased from Innovative Research (SKU#ISERS10ML and SKU#IPLASK2E10ML) to compare results from our analyses across sample preparations. While plasma is produced when whole blood is collected in tubes that are treated with anticoagulant, serum is obtained after allowing blood to clot for 30 minutes at room temperature and then centrifuging the samples to remove the cellular component (2, 3). Studies demonstrate that cellular components significantly increase in samples that sit longer than 60 minutes while clotting; however, adherence to standard operating procedures for preparation of serum and plasma have been found to greatly reduce contamination and sources of error (4). We took extra steps to address these concerns by ensuring timely processing of blood

samples and performing an additional bead purification after cfDNA isolation to remove high-molecular weight DNA, likely derived from contaminating blood cell lysis (**Supplemental Figure 13A and B**). We found that taking this approach, cfDNA methylation status at the block level is highly correlated when comparing cfDNA derived from serum or plasma (**Supplemental Figure 13C**; Pearson r = 0.95). In addition, deconvolution analysis verified that the %immune and %solid organ origins of cfDNA does not vary across the two sample types (**Supplemental Figure 13D**). In fact, there appears to be slightly less variation across donors in the predicted cell type proportions composing cfDNA extracted from serum compared to plasma. Thus, despite an overall higher Geq Immune found in serum due to the overall higher cfDNA concentrations, this background signal is consistent from sample-to-sample allowing for accurate comparison of changes over time in serial samples collected from the same individuals (**Supplemental Figure 13D and E**).

*RNA isolation, RNA-sequencing, and RT-qPCR analysis*

RNA was isolated from tissues or sorted cells using the RNeasy Kit (Qiagen) following homogenization using the MagNA Lyser (Roche) according to the manufacturer's protocol and quantified by Qubit RNA BR assay (Thermo Fisher Scientific). Total RNA was validated using an Agilent RNA 6000 nano assay on the 2100 Bioanalyzer TapeStation (Agilent Technologies). The resulting RNA Integrity number (RIN) of samples selected for downstream qPCR or RNAseq analysis was at least 7. Reverse transcription (RT) was done using the iScript cDNA Synthesis Kit (Bio-Rad) according to the manufacturer's protocol. Real-time quantitative RT–PCR was performed with iQ SYBR Green Supermix

(Bio-Rad). Primers used for RT–qPCR were purchased from Integrated DNA Technologies, and their sequences are provided in **Supplemental Table 9**. Fold change was calculated as a percentage normalized to housekeeping gene actin (*Actb*) using the delta-Ct method. All RT–qPCR assays were done in triplicate. RNA-sequencing libraries were prepared using TruSeq Total RNA library Prep Kit (Illumina) at Novogene Corporation Inc., and 150bp paired-end sequencing was performed on an Illumina HiSeq 4000 with a depth of 50 million reads per sample. A reference index was generated using GTF annotation from GENCODEv28. Raw FASTQ files were aligned and assembled to GRCh38 and GRCh37 with HISAT2 / Stringtie (V 2.1.0) (5). The differential expression was analyzed in R with packages EdgeR (V 3.32.1) and Rsubread (V1.6.3) (6, 7). Derived counts per million and p-values were used to create a rank ordered list, which was then used for subsequent integrative analysis. Expression levels at known cell type markers from single cell expression databases were used to validate the identity of isolated cell type populations for methylome analysis (8).

*Reference DNA methylation data from healthy tissues and cells*

Controlled access to reference WGBS data from normal human tissues and cell types was requested from public consortia participating in the International Human Epigenome Consortium (IHEC) (9) and upon approval downloaded from the European GenomePhenome Archive (EGA), Japanese Genotype-phenotype Archive (JGA), database of Genotypes and Phenotypes (dbGAP), and ENCODE portal data repositories (**Supplemental Table 1**) (10–12). Reference mouse WGBS data from normal tissues and cells were downloaded from selected GEO and SRA datasets (**Supplemental Table 2**)

(13–26). We required that all reference methylomes included for analysis be sequencing libraries made from bisulfite-converted DNA that we could process starting from the raw sequencing data. Downloaded FASTQs were processed and realigned in a similar manner as the locally generated bisulfite-sequencing libraries described above. However, parameters were adjusted to account for each respective WGBS library type. Methylation bias was assessed using the diagnostic tool as part of the Bismark package and read 5' and 3' trimming, mapping, and deduplication were performed based on the recommendations in the Bismark user guide for working with different library types and commercial kits (http://felixkrueger.github.io/Bismark/Docs/). WBGS libraries were deduplicated using deduplicate_bismark (V 0.22.3) All studies used purified DNA from cells or tissues as starting material, except for a subset of immune cell WGBS data generated by Blueprint epigenomics that performed bisulfite conversion directly on lysed cells (µWGBS protocol). Special consideration of bisulfite conversion efficiency was given to samples prepared by the µWGBS protocol and reads with a bisulfite conversion rate below 90% or with fewer than three cytosines outside a CpG context were removed (27). For these cell-types with multiple data sources, we provide additional results in **Supplemental Figures 2 and 3** to show that samples cluster by cell-type and not by library preparation method when performing unsupervised clustering analysis of the top ~30,000 variable blocks amongst all of the samples in the human and mouse datasets, respectively. Of note, for the reference methylation data that we generated ourselves in the lab, we validated the identity of the starting cell population through either RNA-sequencing or FACS analysis. The human cardiopulmonary- and liver sinusoidal-endothelial methylation sequencing data that we generated clustered closely with liver

endothelial data generated by a separate study (25) and with HUVEC methylation data generated by Blueprint Epigenomics (EGAD00001002294). Likewise, the mouse immune cell-type methylation data that we generated clustered closely with other Bcell (17) and Tcell WGBS data (15) generated by other studies.

*Identification of cell-type specific methylation blocks*

We reduced the original 297 human WGBS samples to a final set of 104 samples to identify differentially methylated cell-type specific blocks. First, samples were split into training and testing groups for model validation (80% train and 20% test). The unsupervised hierarchical clustering analysis in **Figure 1** and **Supplemental Figure 1** were performed using the training human and mouse WGBS data detailed in **Supplemental Tables 1 and 2** through being assigned to a UMAP group (Column C). We further excluded samples from bulk tissues and those that did not have sufficient coverage (missing values in >50% of methylation blocks). Outlier replicates, or those clustering with fibroblasts or stromal cell types were excluded, due to possible contamination. Only immune cell methylomes that were reprocessed from raw sequencing data to PAT files were used to identify DMBs. We organized the final 104 human reference samples into groupings of 20 cell types (**Supplemental Table 1**). Similarly, the starting 109 mouse WGBS samples were reduced to a final set of 43 samples that were organized into a final grouping of 9 cell types and tissues (**Supplemental Table 2**). The final samples used to identify DMBs are designated by having an ‘X’ in Column E titled “Included in Atlas” in **Supplemental Tables 1 and 2**.

Tissue and cell-type specific methylation blocks were identified from reference WGBS data using custom scripts (**Supplemental code**). We performed a one-vs-all comparison to identify differentially methylated blocks unique for each group. This was done separately for human and mouse. From this we first identified blocks covering a minimum of three CpG sites, with length less than 2Kb and at least 10 observations. Then, we calculated the average methylation per block/sample, as the ratio of methylated CpG observations across all sequenced reads from that block. Differential blocks were sorted by the margin of separation, termed "delta-beta", defined as the minimal difference between the average methylation in any sample from the target group vs all other samples. Then, we computed the "soft margin" between target samples and background samples, allowing for some outliers using percentiles (**Supplemental Figure 15**). For all markers we calculated the difference between the 80$^{th}$ percentile of the methylation status in the target group (target.quant) and the 10$^{th}$ percentile of the methylation status in the background group (bg.quant). We selected blocks with a soft margin $\geq$ 0.4 for human and $\geq$ 0.35 for mouse. This resulted in a variable number of cell-type specific blocks available for each tissue and cell type. Blocks with a (-) direction are hypomethylated and (+) direction are hypermethylated, defined as a as a direction of methylation in the target cell-type relative to all other tissues and cell-types included in the atlas. We also used a magnitude threshold where all hypomethylated blocks have an Average Methylation Fraction (AMF) <0.5 and hypermethylated blocks have an AMF >0.5. However, the vast majority of cell-type specific differentially methylated blocks are much more diverged (mean AMF hypo = <10% methylation and mean AMF hyper = > 80%). Identified cell-type specific hypomethylated blocks in humans had a mean AMF of 0.081 $\pm$ 0.082 STDEV

(median = 0.06; mode = 0.0). In mouse, hypomethylated blocks had a mean AMF of 0.079 $\pm$ 0.083 STDEV (median = 0.05; mode = 0.0). Likewise, identified cell-type specific hypermethylated blocks in humans had a mean AMF of 0.83 $\pm$ 0.10 STDEV (median = 0.85; mode = 0.9). In mouse, hypermethylated blocks have a mean AMF of 0.83 $\pm$ 0.083 STDEV (median = 0.85; mode = 0.89).

Additional separation of endothelial cell populations from different tissues was performed to identify unique markers for liver endothelial versus cardiopulmonary endothelial blocks that do not overlap. Separately, pan-endothelial blocks were identified with methylation status in common to all endothelial cell populations. Similarly, individual immune cell-type specific methylation blocks were identified for purified cell populations. In addition, bulk immune blocks were identified with methylation status in common to all hematopoietic cell populations. The bulk immune methylation blocks were found to separate all hematopoietic cell types from solid organ cell types of different lineages and were used for deconvolution in the circulation. The solid organ compartment was then further parsed into individual cell-type contributors as specified in Column F titled “Atlas Groups” in **Supplemental Tables 1 and 2**. For some solid organ cell-types, a reduced subset of blocks (ie. top 200) were used for deconvolution in the circulation if the original number identified was greater than one standard deviation above the mean. The reduced subset of blocks were prioritized based on the margin of separation taking into account the quantile variation amongst all samples in target and background groups as described above. In addition, blocks were prioritized that consistently met our designated thresholds for cell-type specificity following iterative leave-one-out analysis of repeated segmentation and marker selection. Selected human and mouse blocks for cell types of interest that

were used for deconvolution of cell-free DNA in the circulation can be found in **Supplemental Tables 3 and 4.** Extended cell-type specific blocks for purified populations of endothelial and immune cell-types can be found in **Supplemental Tables 8 and 15**. For multiple cell-types in both human and mouse, we analyzed samples sourced from multiple consortiums using different library construction methods and still found the identified cell-type specific DMBs to be representative of all samples (**Supplemental Tables 1 and 2**). In addition, when forming groups to identify cell-type specific DMBs we required at least 3 or more samples be present for each group to ensure DMBs were reproducible across biological replicates.

*Methylation score and visualization of cell-type specific methylation atlas*

Each DNA fragment was characterized as U (mostly unmethylated), M (mostly methylated) or X (mixed) based on the fraction of methylated CpG sites as previously described (28). We used thresholds of ≤33% methylated CpGs for U reads and ≥66% methylated CpGs for M. We then calculated a methylation score for each identified celltype specific block based on the proportion of U/X/M reads among all reads. The U proportion was used to define hypomethylated blocks and the M proportion was used to define hypermethylated blocks. Heatmaps were generated using the pretty heatmap function in the RStudio Package for the R bioconductor (RStudioTeam, 2015).

*In-silico simulations and WGBS deconvolution*

In silico mix-in simulations were performed using *wgbstools* (V 0.1.0) (29) to validate the fragment-level deconvolution algorithm at the identified cell-type specific blocks

(**Supplemental Figures 9, 10, 11 and 12**). Reference data with greater than three replicates per cell type were split into independent training and testing sets, leaving at least one replicate out for testing. Since the mouse lung endothelial reference WGBS data had only three replicates, sequenced fragments were merged across replicates for this cell type and then randomly split into training (80%) and testing (20%) sets (using wgbstools merge and then wgbstools pat_splitter). The cell-type specific blocks included in the human and mouse methylation atlases were constructed using training set fragments only. For each cell type profiled, we mixed known proportions of target fragments into a background of leukocyte fragments using wgbstools mix_pat. The leukocyte fragments were obtained from n=4 buffy coat samples in mouse and n=5 buffy coat samples in human. We performed three replicates for each admixture ratio assessed (0.05%, 0.1%, 0.5%, 1%, 2%, 5%, 10%, 15%), which were analyzed as described above, and present the average predicted proportion and standard deviation across all replicates. Model accuracy was assessed through correct classification of the actual percent target mixed.

Each mixture was analyzed using our WGBS atlas and fragment-level deconvolution model in contrast to the 450K array atlas and NNLS model described in Moss et al(30). Array based 450K data were simulated using wgbstools beta_to_450k function (V0.1.0) and deconvolution performed as in Moss et al. (github.com/nloyfer/meth_atlas). Our sequencing-based approach allowed for fragment-level cfDNA analysis of CpG methylation patterns, as opposed to relying on the use of single CpG sites from methylation array data (31). From the in-silico mix-in simulations, we found that our probabilistic fragment-level deconvolution model

outperforms traditional array-based analysis for each tissue and cell type of interest to validate the prediction accuracy and sensitivity (**Supplemental Figure 12**). However, there are inherent differences in the 450K array meth-atlas and NNLS model that prohibited a direct one-to-one comparison. First, the test WGBS data used to generate the in silico mixed samples has limited depth compared to the effective number of molecules measured by DNA methylation arrays. In addition, the meth-atlas is a complete deconvolution NNLS model and thus target fragments were admixed with leukocyte fragments across all features in the 450K array meth-atlas, which may have had variable coverage at informative features specific for each cell-type. Also, many of the features in the 450K array atlas were identified for tissues and not the purified cell-type WGBS data used in this study (ie. left atrium heart tissue as opposed to cardiomyocytes and lung tissue as opposed to lung epithelial cells). However, we found that pattern analysis at the cell-type specific methylation blocks identified here allowed for accurate detection of cfDNA from a given source when present in less than 0.1% of a mixture, a marked improvement in comparison to current 450K approaches (**Supplemental Figure 12**).

*Functional annotation and pathway analysis*

Cell-type specific methylation blocks were provided as input for analysis in HOMER (V4.11.1) (http://homer.ucsd.edu/homer/) (32). Each block was associated with its closest nearby gene and provided a genomic annotation using the annotatePeaks.pl function, with "-size given -CpG" parameters. By default, TSS (transcription start site) was defined from -1 kb to +100 bp, TTS (transcription termination site) was defined from -100 bp to +1 kb, and CpG islands were defined as a genomic segment with GC content ≥50%, genomic

length >200 bp and the ratio of observed/expected CpG number >0.6. Prediction of known and de-novo transcription factor binding motifs were also assessed by HOMER using the findMotifsGenome.pl function. The top 5 motifs based on p-value were selected from each analysis. Pathway analysis of identified tissue and cell-type specific methylation blocks was performed using Ingenuity Pathway Analysis (IPA) (33) (Qiagen) and Genomic Regions Enrichment of Annotations Tool (GREAT) (34). GeneSetCluster was used to cluster identified gene-set pathways based on shared genes (35). Canonical pathways/functional annotations were grouped into clusters by calculating the similarity of pathways/annotations using the relative risk (RR) of each pathway appearing based on the genes enriched within the pathway. RR scores were clustered into groups using kmeans. Over-representation analysis was implemented in the WebgestaltR (ORAperGeneSet) plugin to interpret and functionally label identified gene-set clusters (36). Integration of methylome and transcriptome data generated from tissue-specific endothelial cells was performed using an expanded set of cell-type specific blocks (--bg.quant 0.2) compared to the more restricted set of blocks used for deconvolution analysis in the circulation (--bg.quant 0.1). The extended endothelial-specific methylation blocks can be found in **Supplemental Table 8**.

*Cross-species comparison and human and mouse cell-type specific DMBs*

For cell-types represented in both human and mouse atlases we performed cross-species mapping using the UCSC Genome Browser LiftOver tool and the hg19ToMm9.over.chain.gz file. Only 24% of human cell-type specific blocks were mapped completely to the mouse genome, with the majority of human blocks having been

deleted or partially deleted in the mm9 genome. The lower mapability rate of epigenetic regions (mostly within introns and intergenic regions) has been demonstrated by others (37, 38). This also reflects previous findings that only 40% of the nucleotides in the human genome can be aligned to mouse (39) and also the difference that there are only ~21 million CpG sites in the mouse mm9 genome relative to the ~28 million CpG sites in the human hg19 genome. Of the blocks that fully mapped, 100% were captured on both of the separately designed human and mouse hybridization capture panels. On average, 18% of these captured and mapped regions were identified as being cell-type specific methylation blocks in both species (**Supplemental Table 14**). Of note, 100% of overlapping cell-type specific methylation blocks had the same methylation status in both human and mouse (ie. human hepatocyte-specific hypomethylated blocks were hypomethylated specifically in mouse hepatocytes too). To circumvent limited cross-species mapability, we also compared overlapping genes associated with identified cell-type specific DMBs in cell-types represented in both human and mouse atlases (**Supplemental Table 14**). However, this is likely an underestimate due to limited overlap of regions captured from the separate human and mouse hybridization panels utilized. We expanded on analysis of genomic distribution to compare the distribution of hypo- and hyper- methylated percentages between human and mouse species on a cell/organ basis. We depict the distribution of cell-specific hypo- and hyper- methylated DMBs on a cell/organ basis in **Supplemental Figure 5**. Within all hypomethylated blocks, there wasn't a significant difference in genomic distribution for the same cell-type between human and mouse species, taking into account the distribution differences at baseline comparing captured regions (Chi-square, df=4, $p>0.05$). The same was true within

hypermethylated blocks (Chi-square, df=4, $p>0.05$). However, there were differences in overall hypo- and hyper- methylated percentages between human and mouse samples across cell-types. Interestingly, all solid organ derived cell-type specific DMBs in mouse were hypomethylated, whereas all immune-cell specific DMBs were hypermethylated. In contrast, most human cell-types had both hypo- and hyper- methylated DMBs identified, with the large majority being hypomethylated. Despite this difference, the human bulk immune cell-type specific DMBs were 47% hypermethylated, suggesting that a higher percentage of hypermethylated markers is common to cell types comprising hematopoietic lineages in both human and mouse species. This was further supported by the 30% of immune cell-specific methylation blocks that overlapped between the human and mouse capture panels that were 100% correlated in methylation status and found to be hypermethylated in both species (**Supplemental Table 14**).

## Supplemental References

1. Maggi EC, et al. Development of a Method to Implement Whole-Genome Bisulfite Sequencing of cfDNA from Cancer Patients and a Mouse Tumor Model. *Frontiers Genetics* 2018;09:6.

2. Kerachian MA, et al. Guidelines for pre-analytical conditions for assessing the methylation of circulating cell-free DNA. *Clin Epigenetics* 2021;13(1):193.

3. Holdenrieder S, et al. Cell-Free DNA in Serum and Plasma: Comparison of ELISA and Quantitative PCR. *Clin Chem* 2005;51(8):1544–1546.

4. Tuck MK, et al. Standard Operating Procedures for Serum and Plasma Collection: Early Detection Research Network Consensus Statement Standard Operating Procedure Integration Working Group. *J Proteome Res* 2009;8(1):113–117.

5. Kim D, Langmead B, Salzberg SL. HISAT: a fast spliced aligner with low memory requirements. *Nat Methods* 2015;12(4):357–360.

6. Robinson MD, McCarthy DJ, Smyth GK. edgeR: a Bioconductor package for differential expression analysis of digital gene expression data. *Bioinformatics* 2010;26(1):139–140.

7. Liao Y, Smyth GK, Shi W. The R package Rsubread is easier, faster, cheaper and better for alignment and quantification of RNA sequencing reads. *Nucleic Acids Res* 2019;47(8):e47–e47.

8. Khan S, et al. EndoDB: a database of endothelial cell transcriptomics data. *Nucleic Acids Res* 2018;47(Database issue):gky997-.

9. Bujold D, et al. The International Human Epigenome Consortium Data Portal. *Cell Syst* 2016;3(5):496-499.e2.

10. Dunham I, et al. An integrated encyclopedia of DNA elements in the human genome. *Nature* 2012;489(7414):57–74.

11. Davis CA, et al. The Encyclopedia of DNA elements (ENCODE): data portal update. *Nucleic Acids Res* 2017;46(Database issue):gkx1081-.

12. Consortium RE, et al. Integrative analysis of 111 reference human epigenomes. *Nature* 2015;518(7539):317.

13. Sabbagh MF, et al. Transcriptional and epigenomic landscapes of CNS and nonCNS vascular endothelial cells. *Elife* 2018;7:e36187.

14. Schlereth K, et al. The transcriptomic and epigenetic map of vascular quiescence in the continuous lung endothelium. *Elife* 2018;7:e34423.

15. Delacher M, et al. Genome-wide DNA methylation landscape defines specialization of regulatory T cells in tissues. *Nat Immunol* 2017;18(10):1160–1172.

16. dos Santos CO, et al. An Epigenetic Memory of Pregnancy in the Mouse Mammary Gland. *Cell Reports* 2015;11(7):1102–1109.

17. Duncan CG, et al. Base-Resolution Analysis of DNA Methylation Patterns Downstream of Dnmt3a in Mouse Naïve B Cells. *G3 Genes Genomes Genetics* 2018;8(3):805–813.

18. Gilsbach R, et al. Distinct epigenetic programs regulate cardiac myocyte development and disease in the human heart in vivo. *Nat Commun* 2018;9(1):391.

19. Gilsbach R, et al. Dynamic DNA methylation orchestrates cardiomyocyte development, maturation and disease. *Nat Commun* 2014;5(1):5288.

20. Gravina S, et al. Single-cell genome-wide bisulfite sequencing uncovers extensive heterogeneity in the mouse liver methylome. *Genome Biol* 2016;17(1):150.

21. Dong X, et al. Global, integrated analysis of methylomes and transcriptomes from laser capture microdissected bronchial and alveolar cells in human lung. *Epigenetics* 2018;13(3):1–22.

22. Hon GC, et al. Epigenetic memory at embryonic enhancers identified in DNA methylation maps from adult mouse tissues. *Nat Genet* 2013;45(10):1198–1206.

23. Lagger S, et al. MeCP2 recognizes cytosine methylated tri-nucleotide and dinucleotide sequences to tune transcription in the mammalian brain. *Plos Genet* 2017;13(5):e1006793.

24. Pidsley R, et al. Critical evaluation of the Illumina MethylationEPIC BeadChip microarray for whole-genome DNA methylation profiling. *Genome Biol* 2016;17(1):208.

25. Jamil MA, et al. Molecular Analysis of Fetal and Adult Primary Human Liver Sinusoidal Endothelial Cells: A Comparison to Other Endothelial Cells. *Int J Mol Sci* 2020;21(20):7776.

26. Cheng THT, et al. Noninvasive Detection of Bladder Cancer by Shallow-Depth Genome-Wide Bisulfite Sequencing of Urinary Cell-Free DNA for Methylation and Copy Number Profiling. *Clin Chem* 2019;65(7):927–936.

27. Farlik M, et al. DNA Methylation Dynamics of Human Hematopoietic Stem Cell Differentiation. *Cell Stem Cell* 2016;19(6):808–822.

28. Loyfer N, et al. A DNA methylation atlas of normal human cell types. *Nature* 2023;613(7943):355–364.

29. Loyfer N, Kaplan T. wgbstools: A computational suite for DNA methylation sequencing data representation, visualization, and analysis. *wgbstools* https://github.com/nloyfer/wgbs_tools. (accessed 25 May 2023).

30. Moss J, et al. Comprehensive human cell-type methylation atlas reveals origins of circulating cell-free DNA in health and disease. *Nat Commun* 2018;9(1):5068.

31. Li W, et al. CancerDetector: ultrasensitive and non-invasive cancer detection at the resolution of individual reads using cell-free DNA methylation sequencing data. *Nucleic Acids Res* 2018;46(15):e89–e89.

32. Heinz S, et al. Simple Combinations of Lineage-Determining Transcription Factors Prime cis-Regulatory Elements Required for Macrophage and B Cell Identities. *Mol Cell* 2010;38(4):576–589.

33. Krämer A, et al. Causal analysis approaches in Ingenuity Pathway Analysis. *Bioinformatics* 2014;30(4):523–530.

34. McLean CY, et al. GREAT improves functional interpretation of cis-regulatory regions. *Nat Biotechnol* 2010;28(5):495–501.

35. Ewing E, et al. GeneSetCluster: a tool for summarizing and integrating gene-set analysis results. *Bmc Bioinformatics* 2020;21(1):443.

36. Liao Y, et al. WebGestalt 2019: gene set analysis toolkit with revamped UIs and APIs. *Nucleic Acids Res* 2019;47(W1):W199–W205.

37. Klughammer J, et al. Comparative analysis of genome-scale, base-resolution DNA methylation profiles across 580 animal species. *Nat Commun* 2023;14(1):232.

38. Rademacher K, et al. Evolutionary Origin and Methylation Status of Human Intronic CpG Islands that Are Not Present in Mouse. *Genome Biol Evol* 2014;6(7):1579–1588.

39. Breschi A, Gingeras TR, Guigo R. Comparative transcriptomics in human and mouse. *Nat Rev Genet.* 2017;18(7):425-440.

# Supplemental Figure 1.

A Mouse Methylation Reference Data

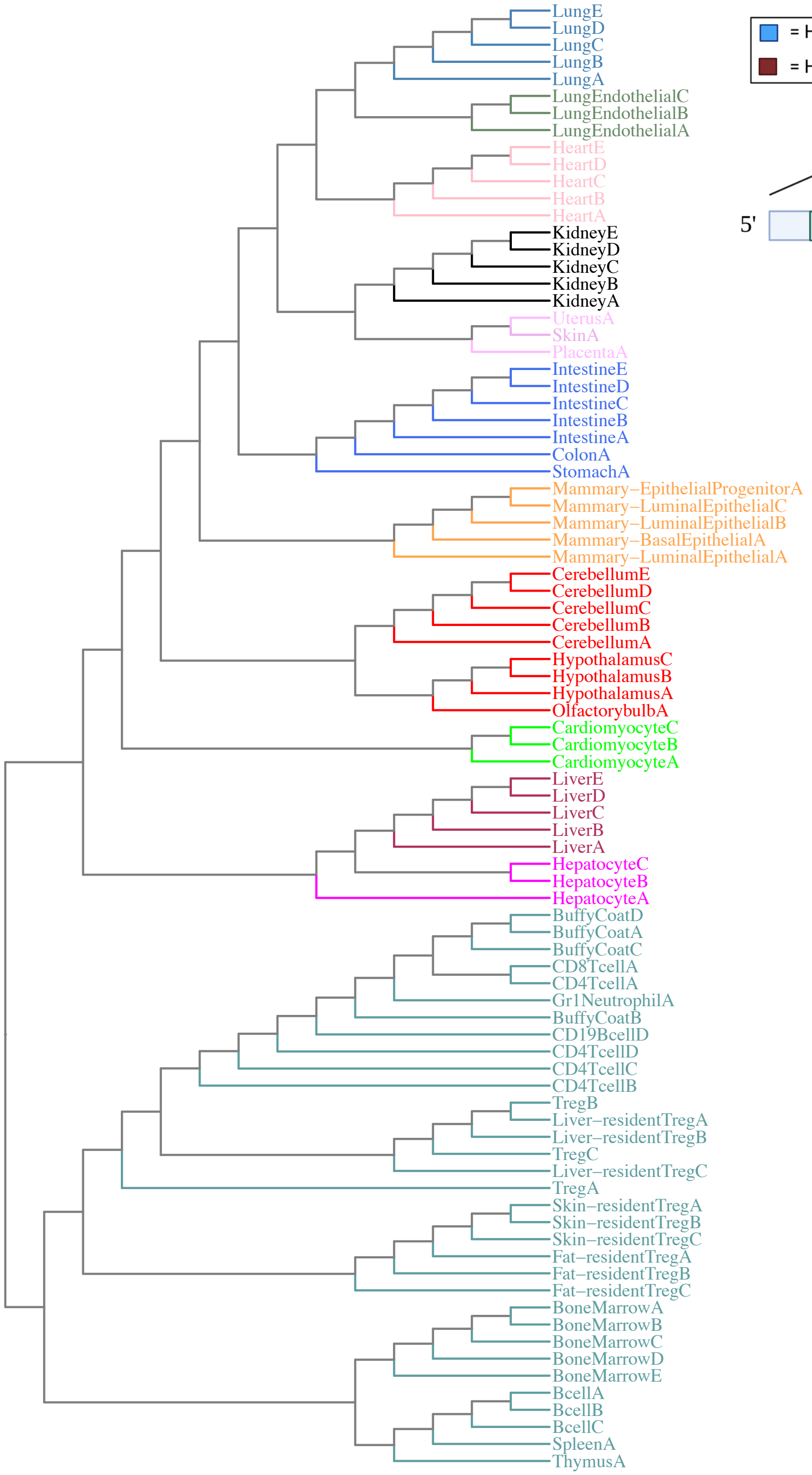


B

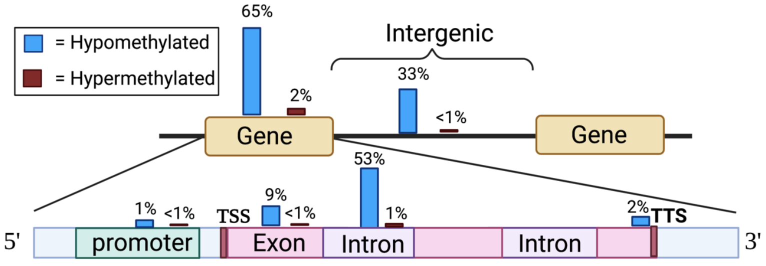

## Supplemental Figures and Legends

**Supplemental Figure 1. Characterization of mouse cell-type specific reference methylation data.** (A) Tree dendrogram depicting relationship between mouse reference Whole Genome Bisulfite Sequencing (WGBS) datasets from different tissues and cell types included in the analysis. Methylation status of the top 30,000 variable blocks across all samples was used as input data for the unsupervised hierarchical clustering. (B) Schematic diagram depicting location of mouse cell-type specific hypo- and hyper-methylated blocks. Genomic annotations of cell-type specific methylation blocks were determined by analysis using HOMER.

# Supplemental Figure 2.

A

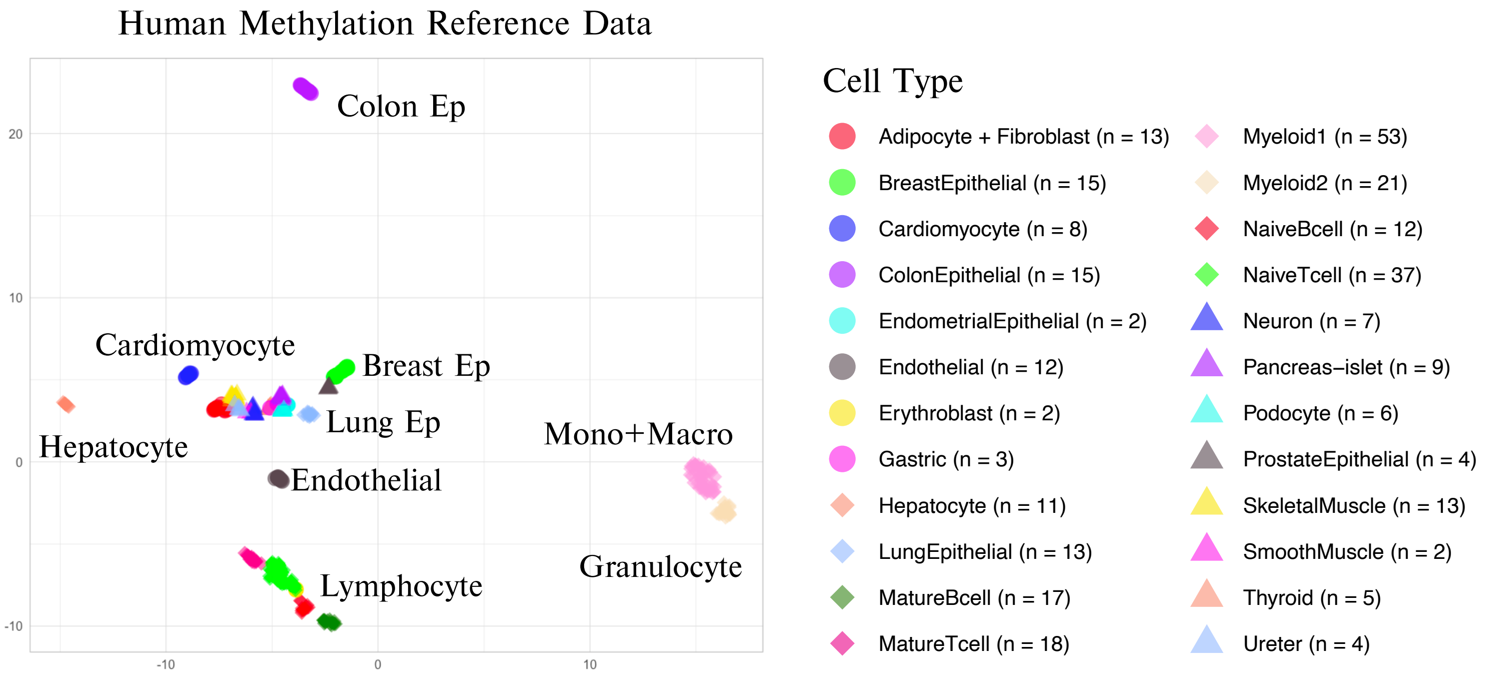


B

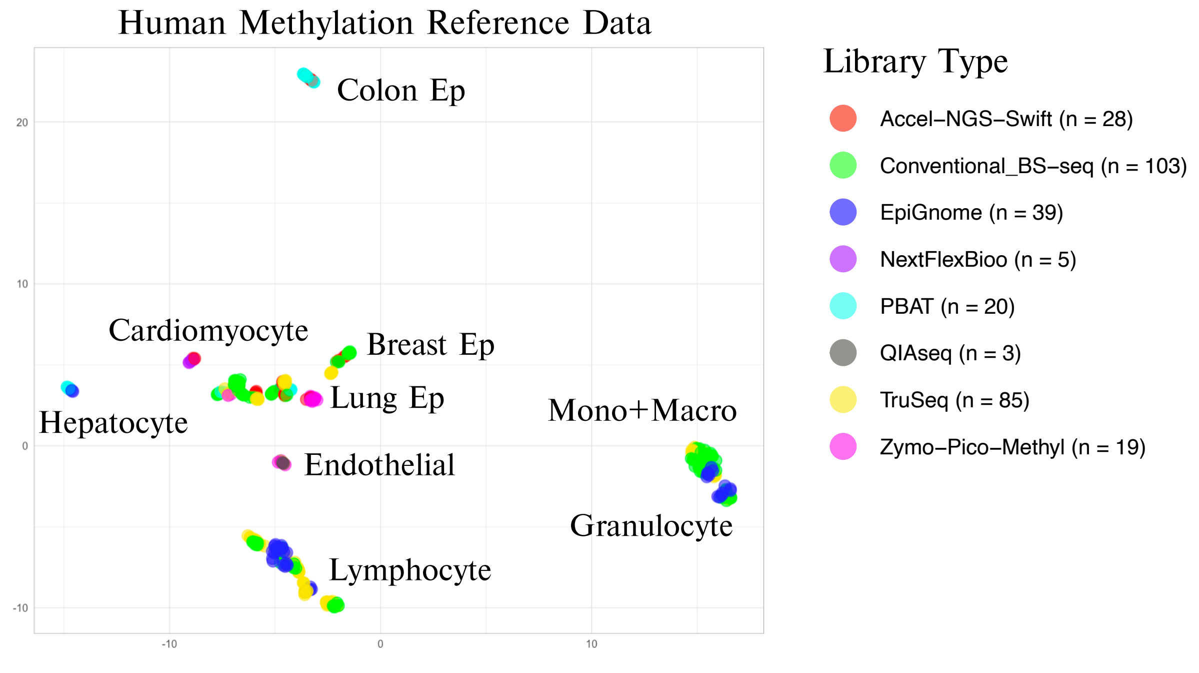

**Supplemental Figure 2. Unsupervised clustering of human cell-type specific reference methylation data reflects developmental lineage.** (A, B) Average methylation was calculated within blocks of at least three CpGs and the top 30,000 blocks were selected showing the highest variability across all samples. UMAP coordinates of the human reference WGBS samples were clustered based on similarity in methylation status at these highly variable blocks. Colors indicate cell type group in (A) and library construction methodology in (B). This analysis systematically grouped biological samples of the same cell type and library construction method did not influence clustering. Abbreviations: CAEC = coronary artery endothelial cell, CMEC = cardiac microvascular endothelial cell, CPEC = joint cardio-pulmonary endothelial cell, HUVEV = human umbilical vein endothelial cell, LSEC = liver sinusoidal endothelial cell, MK = megakaryocyte, NK = natural killer cell, PAEC = pulmonary artery endothelial cell, PMEC = pulmonary microvascular endothelial cell.

# Supplemental Figure 3.

**A**

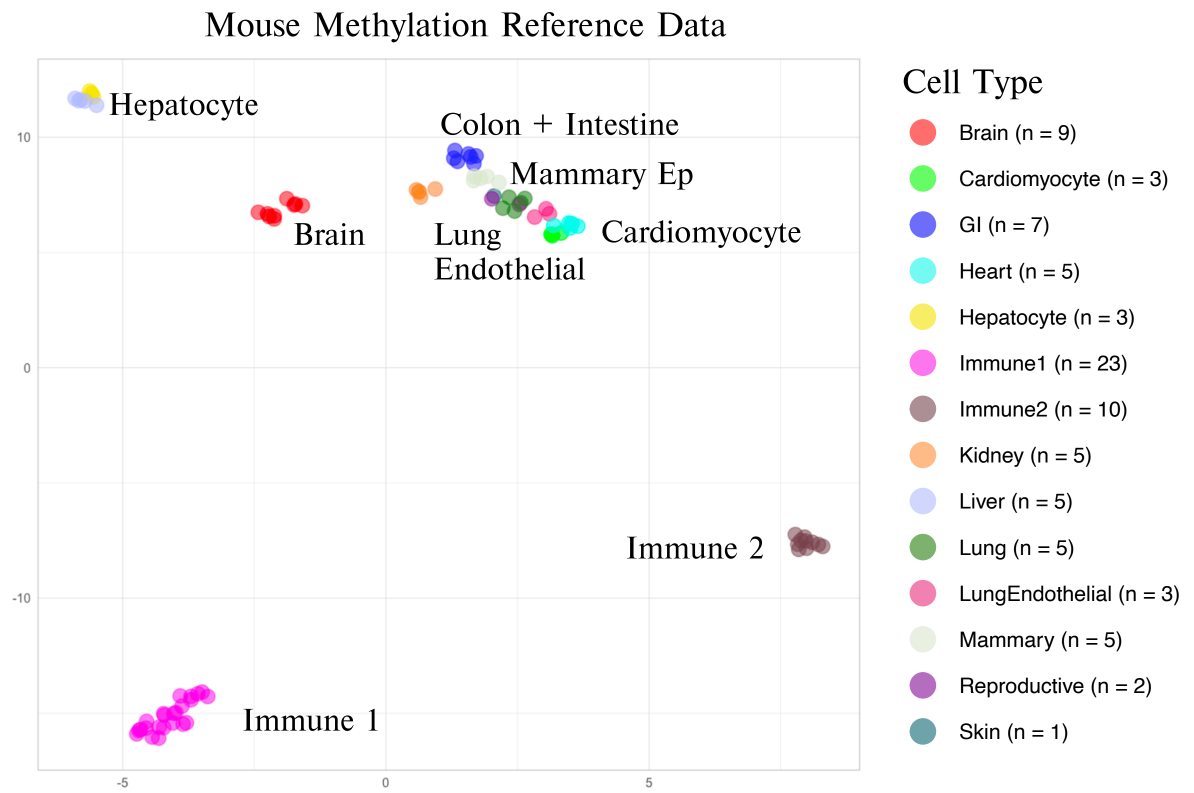


**B**

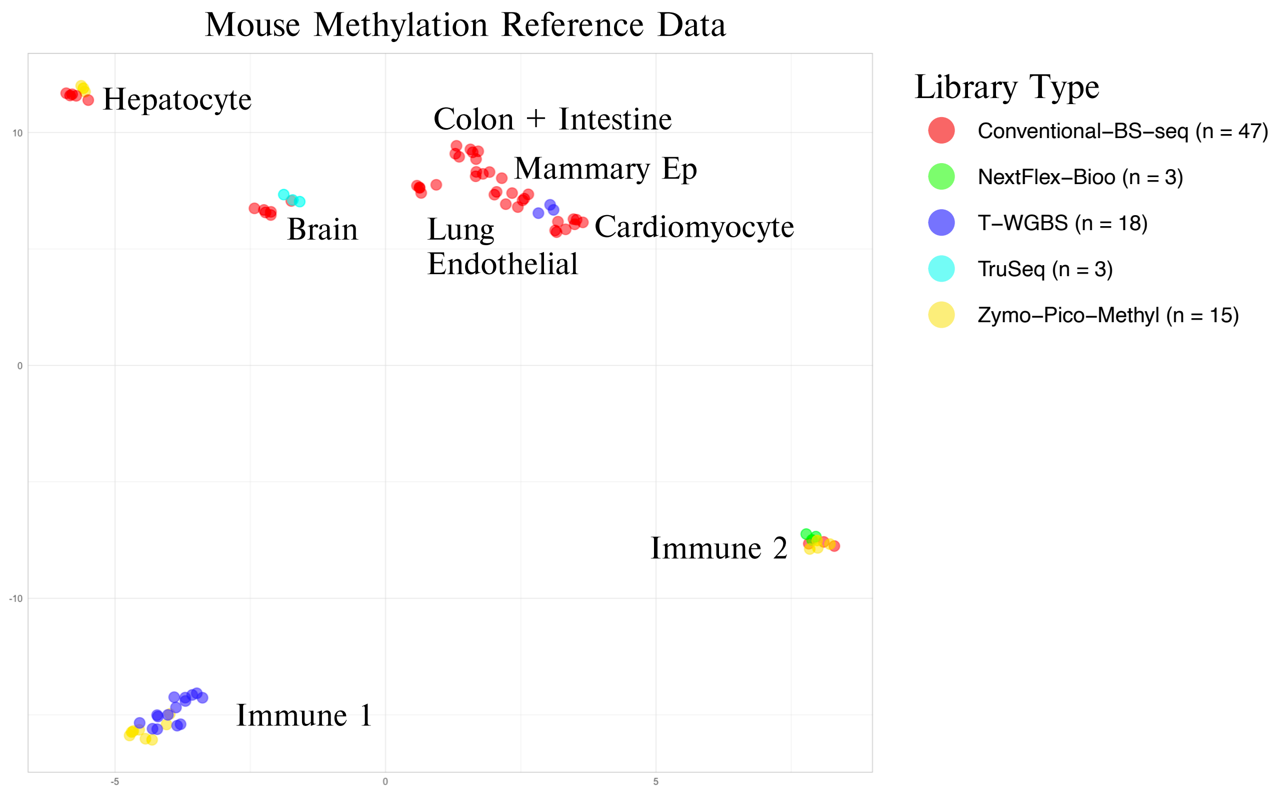

**Supplemental Figure 3. Unsupervised clustering of mouse cell-type specific reference methylation data reflects developmental lineage.** (A, B) Average methylation was calculated within blocks of at least three CpGs and the top 30,000 blocks were selected showing the highest variability across all samples. UMAP coordinates of the mouse reference WGBS samples were clustered based on similarity in methylation status at these highly variable blocks. Colors indicate cell type group in (A) and library construction methodology in (B). This analysis systematically grouped biological samples of the same cell type and library construction method did not influence clustering. Abbreviations: GI = colon + intestine, Immune1 = hematopoietic cell types, Immune2 = lymphoid tissues (bone marrow, spleen, and thymus).

# Supplemental Figure 4.

A

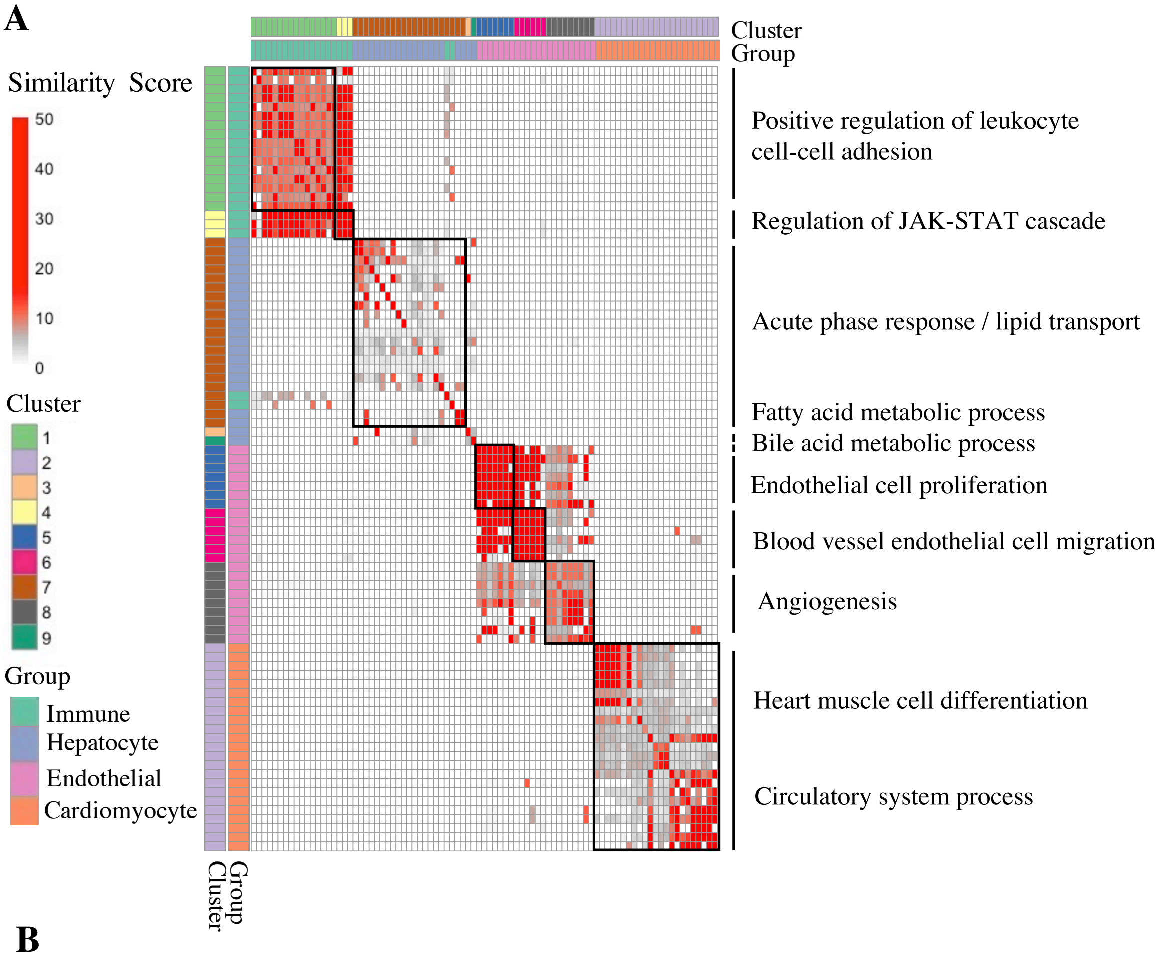


B

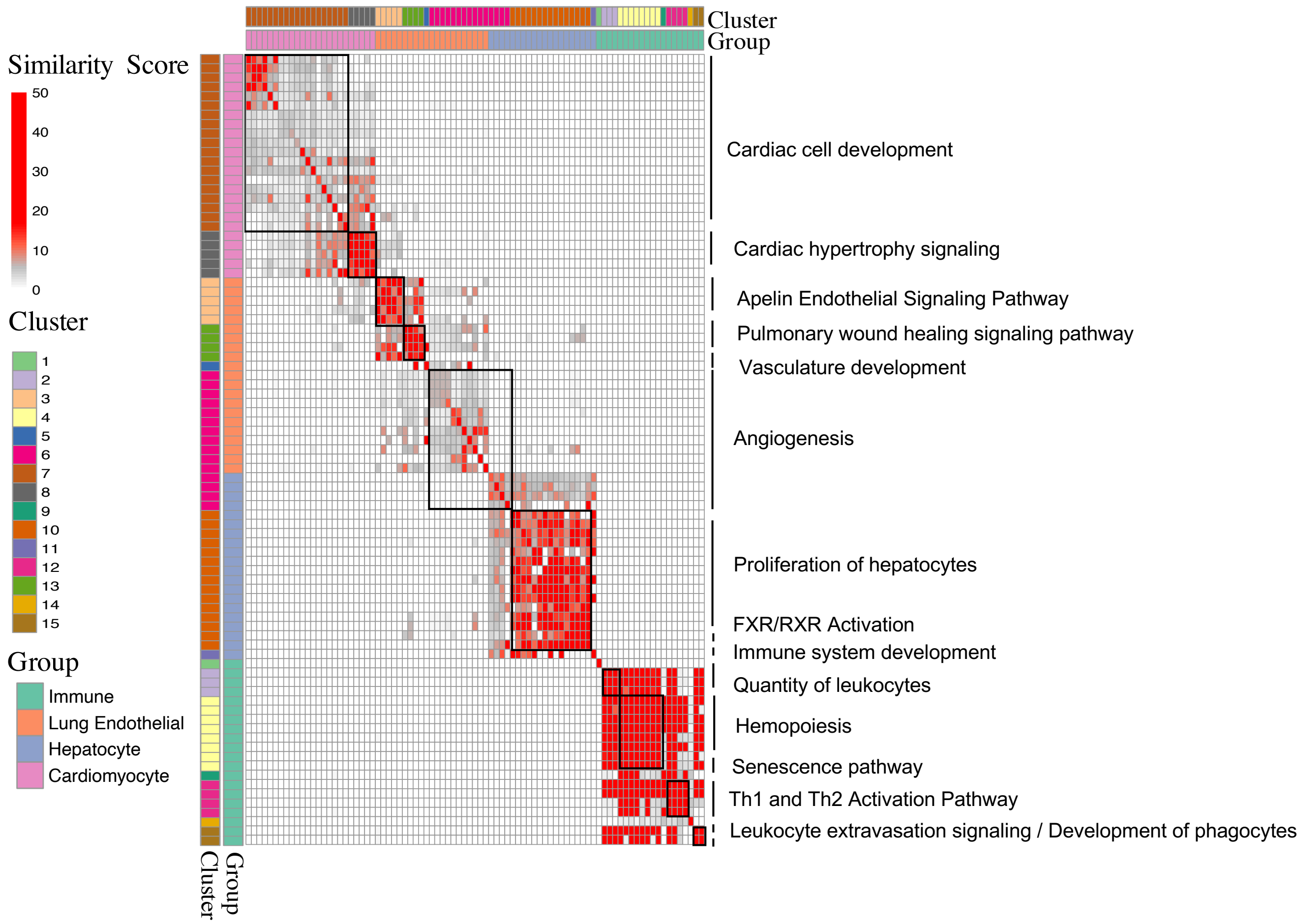

**Supplemental Figure 4. Biological validation of cell-type specific DNA methylation blocks in human and mouse.** (A, B) Heatmap of distance scores between gene-set pathways identified from GeneSetCluster. Genes adjacent to human (A) and mouse (B) cell-type specific methylation blocks were identified using HOMER and pathway analysis was performed using both Ingenuity Pathway Analysis (IPA) and GREAT. Significantly enriched gene-set pathways ($p<0.05$) from differentially methylated blocks identified in immune, cardiomyocyte, hepatocyte, and endothelial cell types were analyzed using GeneSetCluster. Cluster analysis was performed to determine the distance between all identified gene-set pathways based on the degree of overlapping genes from each individual gene set compared to all others. Over-representation analysis was implemented in the WebgestaltR (ORAperGeneSet) plugin to interpret and functionally label identified geneset clusters.

# Supplemental Figure 5.

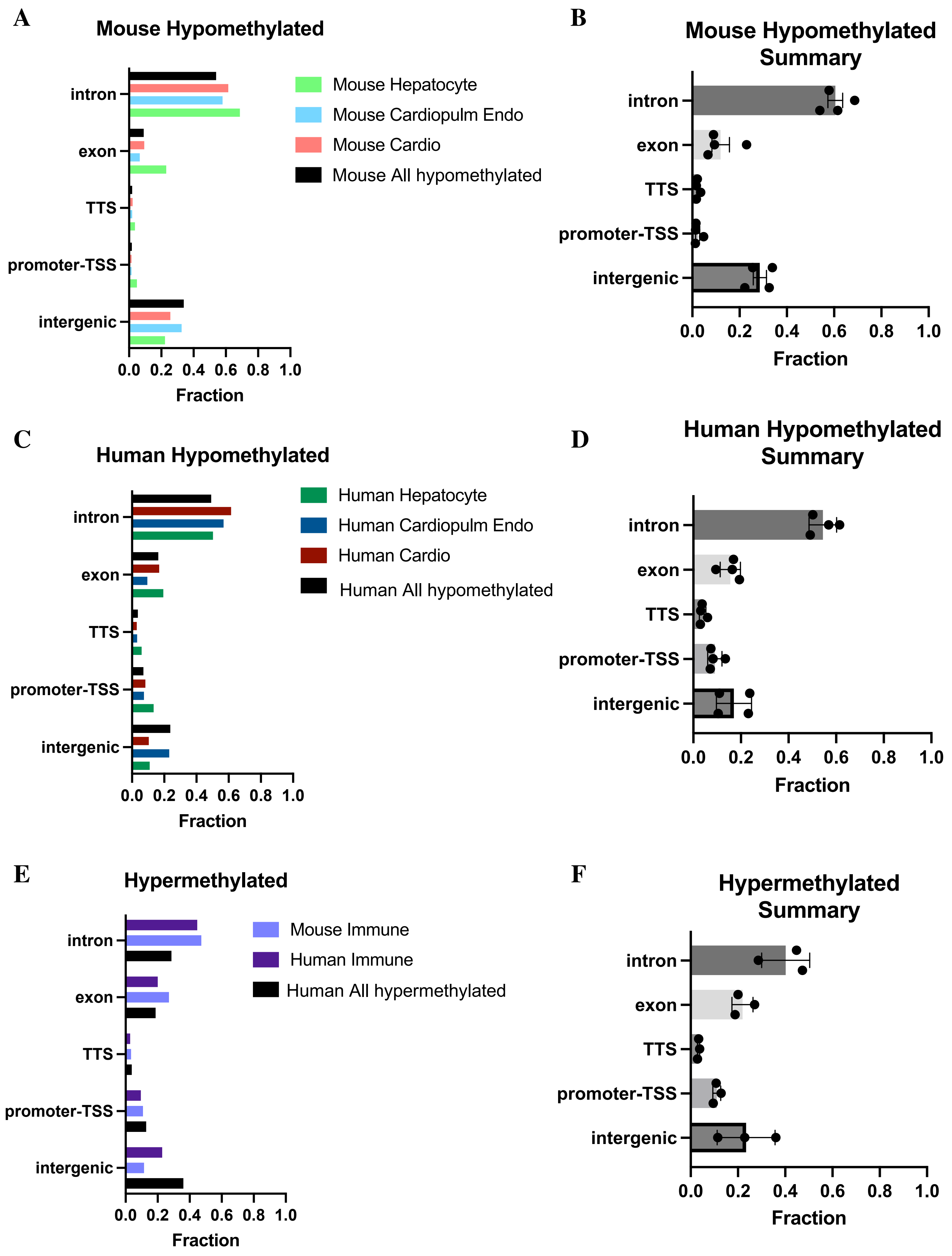

**Supplemental Figure 5. Genomic distribution of human and mouse cell-type specific methylation blocks.** (A) Distribution of mouse hypomethylated blocks for cell-types in common between the human and mouse atlases compared to the distribution of all mouse hypomethylated cell-type specific blocks (All). (B) Average distribution of hypomethylated cell-type specific blocks shown in A. (C) Distribution of human hypomethylated blocks for cell-types in common between the human and mouse atlases compared to the distribution of all human hypomethylated cell-type specific blocks. (D) Average distribution of hypomethylated cell-type specific blocks shown in C. (E) Distribution of human and mouse hypermethylated blocks for cell-types in common. (F) Average distribution of hypermethylated cell-type specific blocks shown in E. (A-E) Genomic annotations of cell-type specific methylation blocks were determined by analysis using HOMER.

# Supplemental Figure 6.

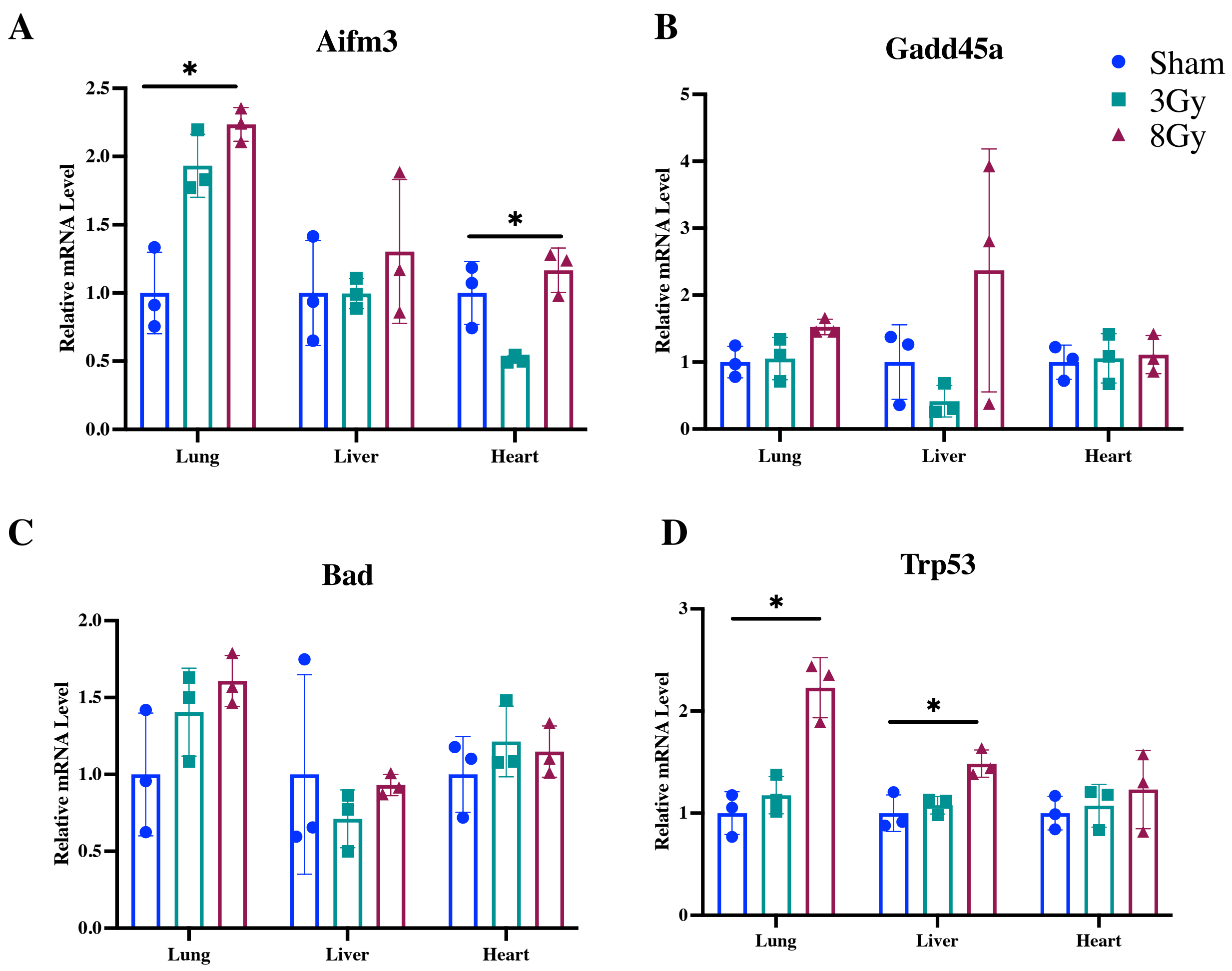

**Supplemental Figure 6. Indicators of damage from radiation in mouse tissues.** qPCR analysis of markers of apoptosis and radiation damage (*Trp53, Gadd45a, Aifm3*, and *Bad*) in mouse lung, heart, and liver tissues treated with 3Gy and 8Gy radiation compared to sham control. The gene expression in each sample was normalized to the expression of *Actb* (beta- actin). Data presented as mean ± SD; N = 3. Kruskal-Wallis test was used for comparisons amongst groups and results were considered significant when *$p < 0.05$; ns, $p \geq 0.05$.

# Supplemental Figure 7.

## Human

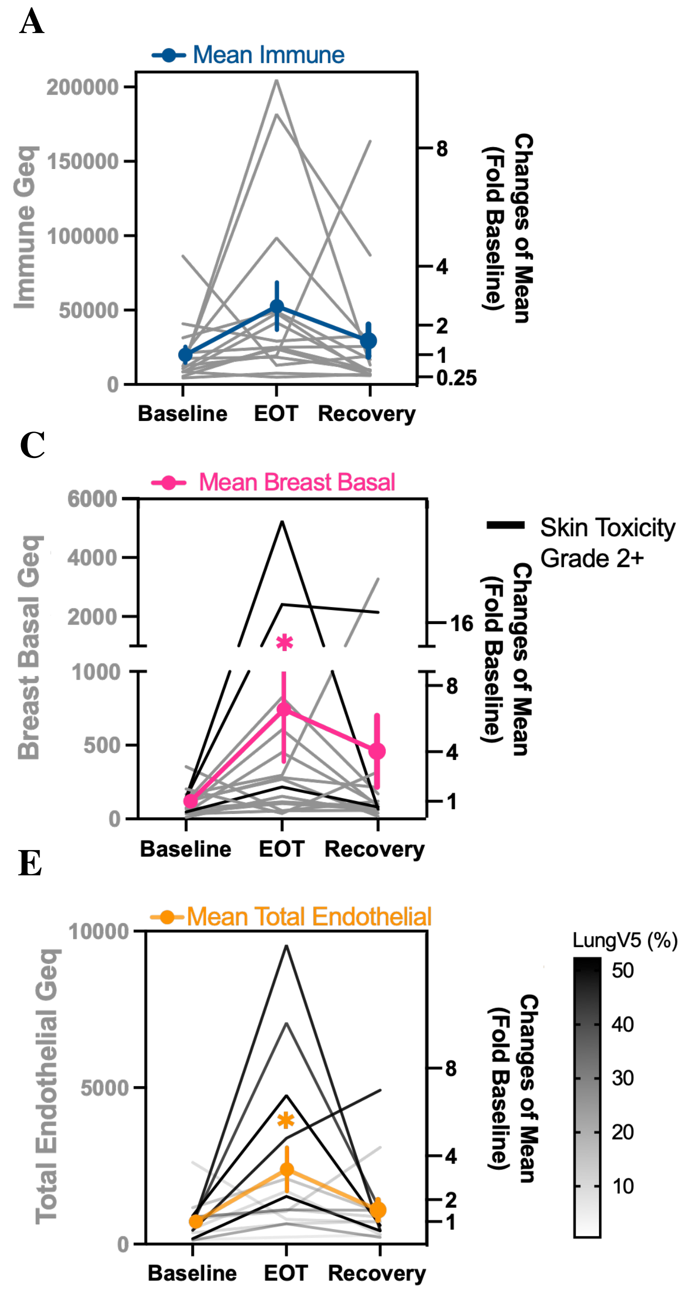


## Mouse

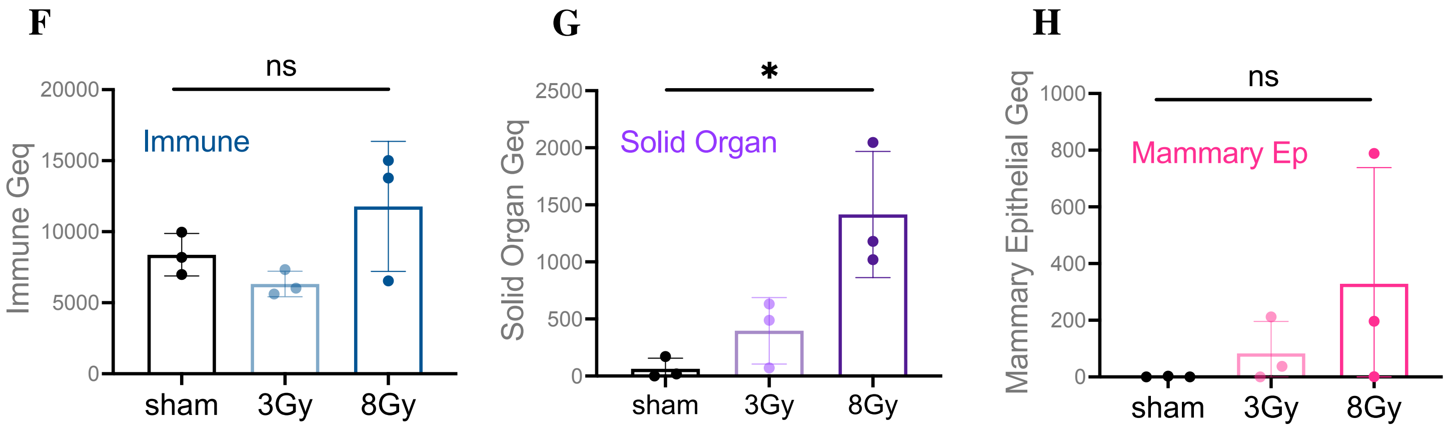

**Supplemental Figure 7. Radiation-induced effects on immune and other solid organ cell-types in human (A-E) and mouse (F-H) samples**. (A) Predicted human immune-derived cfDNA in Geq. Immune cfDNA was assessed at n = 222 methylation blocks found to separate immune cell types from solid organ cell types. (immune = Bcell, CD4Tcell, CD8Tcell, NK, MK, Erythroblast, Monocyte, Macrophage, Neutrophil; solid organ = breast basal/luminal epi, lung epi, hepatocyte, kidney podocyte, pancreas islet, colon epi, cardiomyocyte, LSEC, CPEC, HUVEC, neuron, and skeletal muscle). (B) Predicted human solid organ-derived cfDNA in Geq where % solid organ is defined as 100-%immune using the above n=222 methylation blocks. (C) Breast basal epithelial cfDNA (in Geq/mL). Fragment-level deconvolution used top breast basal epithelial specific methylation blocks (n=200). (D) Breast luminal epithelial cfDNA (in Geq/mL). Fragment-level deconvolution used breast luminal epithelial specific methylation blocks (n=330). (E) Predicted total endothelial cfDNA (in Geq/mL). Fragment-level deconvolution was assessed at n = 131 methylation blocks found to separate endothelial cells from all other cell types. (endothelial = CPEC, LSEC, HUVEC; non-endothelial = Bcell, CD4Tcell, CD8Tcell, NK, MK, Erythroblast, Monocyte, Macrophage, Neutrophil, breast basal/luminal epi, lung epi, hepatocyte, kidney podocyte, pancreas islet, colon epi, cardiomyocyte, neuron, and skeletal muscle). (A-E) Friedman test was performed for comparisons amongst groups. ns, $p \geq 0.05$; *, $p < 0.05$; immune p=0.07, solid organ p=0.008, breast basal epithelial p=0.002, breast luminal epithelial p=0.02, total endothelial p=0.01. Mean fold change relative to baseline is presented as mean ± SEM; N = 15. (F) Predicted mouse immune-derived cfDNA in Geq. Immune cfDNA was assessed at n = 148 methylation blocks found to separate immune cell types from solid organ cell types. (immune = Bcell, CD4Tcell, CD8Tcell, Neutrophil; solid organ = mammary epi, cardiomyocyte, hepatocyte, lung endothelial, cerebellum, hypothalamus, colon, intestine, kidney). (G) Predicted mouse solid organ-derived cfDNA (in Geq/mL). (H) Mammary epithelial cfDNA (in Geq/mL). Fragment-level deconvolution used mouse mammary epithelial specific methylation blocks (n=874). (F-H) Mean ± SD; N = 3 independent methylome preparations. Kruskal-Wallis test was used for comparisons amongst groups. ns, $p \geq 0.05$; *, $p < 0.05$; immune p = 0.20, solid organ p = 0.01, mammary epithelial p=0.19.

# Supplemental Figure 8.

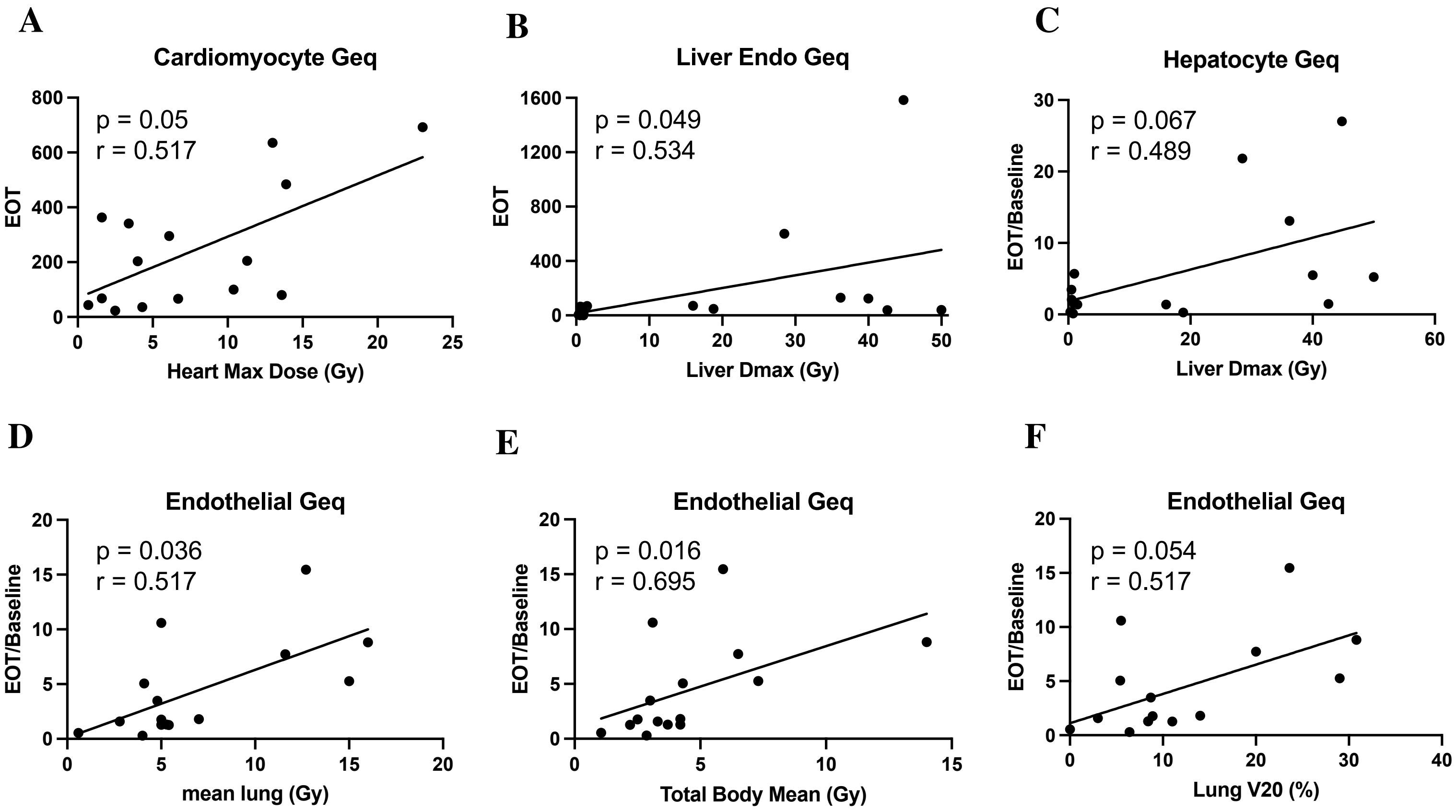

**Supplemental Figure 8. Correlation of methylated DNA in the circulation with human dosimetry data.** (A) Correlation of cardiomyocyte cfDNA at end-of-treatment (EOT) with the maximum dose to the heart (Gy). (B) Correlation of liver endothelial cfDNA at EOT with the maximum dose to the liver (Gy). (C) Correlation of hepatocyte cfDNA with the maximum dose to the liver (Gy). EOT/Baseline represents the fraction of hepatocyte cfDNA post-radiation at end-of-treatment (EOT) relative to baseline levels. (D-F) Correlation of total endothelial cfDNA with the mean dose to the lung (D), total body mean dose (E) and Lung V20 (%). The volume of the lung receiving 20 Gy dose is represented by Lung V20 (%). EOT/Baseline represents the fraction of endothelial cfDNA post-radiation at end-of-treatment (EOT) relative to baseline levels. *(c, e, g) Spearman correlation r was calculated, and linear correlation was considered significant when *$p < 0.05$; ns, $p \geq 0.05$.

# Supplemental Figure 9.

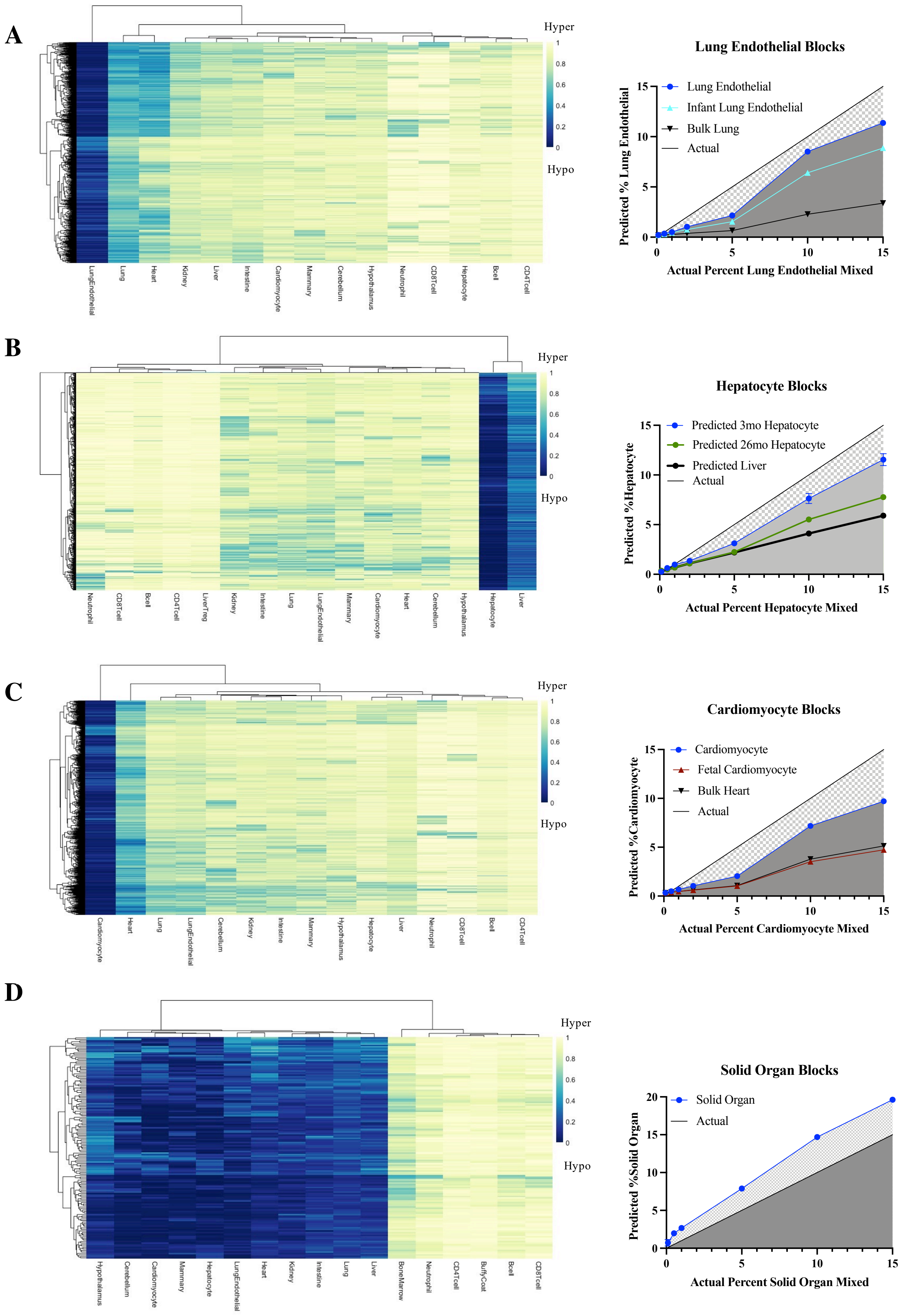

**Supplemental Figure 9. Sensitivity and specificity of identified mouse cell-type specific differentially methylated blocks.** (A-D) (Left) Heatmap of all cell-type specific methylation blocks selected for each target cell type. All blocks contain $\geq$3 CpG sites and have a margin of beta difference greater than or equal to 0.35 separating the target cell type from all others included in the reference maps. All identified methylation blocks for lung endothelial (n=1,546), hepatocyte (n=616), and cardiomyocyte (n=2,917) mouse cell types were hypomethylated. In contrast, all identified immune cell-specific blocks (n=148) were hypermethylated relative to other solid organ cell types in mouse. (Right) In-silico mix-in validation using a fragment-level probabilistic deconvolution model. Target cell-type read-pairs were in-silico mixed into a background of lymphocyte or buffy coat read-pairs at various known percentages (0.5%, 1%, 2%, 5%, 10%, 15%). The deconvolution model was validated on these in-silico mixed samples of known celltype proportions at the blocks selected. The average predicted %target is shown relative to the known %mixed to assess sensitivity and specificity of the identified cell-type specific blocks and deconvolution model. Data presented as mean ± SD; N=3 replicates per proportion. Reference WGBS samples with less than 3 replicates were split into "0.8 train" to select methylation blocks and "0.2 test" to generate in-silico mixed samples. When available, in-silico mixed samples of the same cell type derived from differently aged mice were also tested (infant < 6 weeks old). In addition, bulk tissue containing the respective cell type was tested as well.

# Supplemental Figure 10.

A

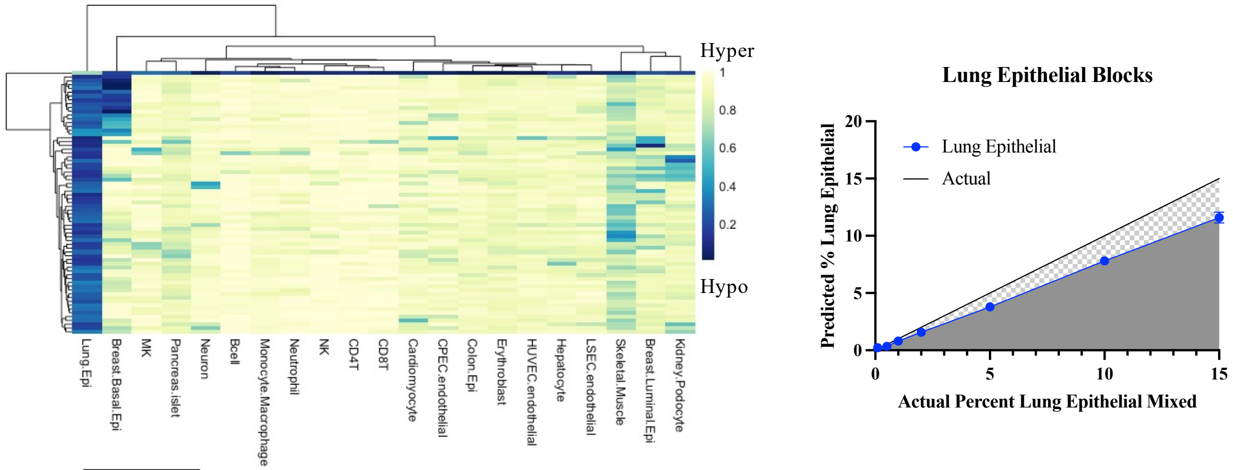


B

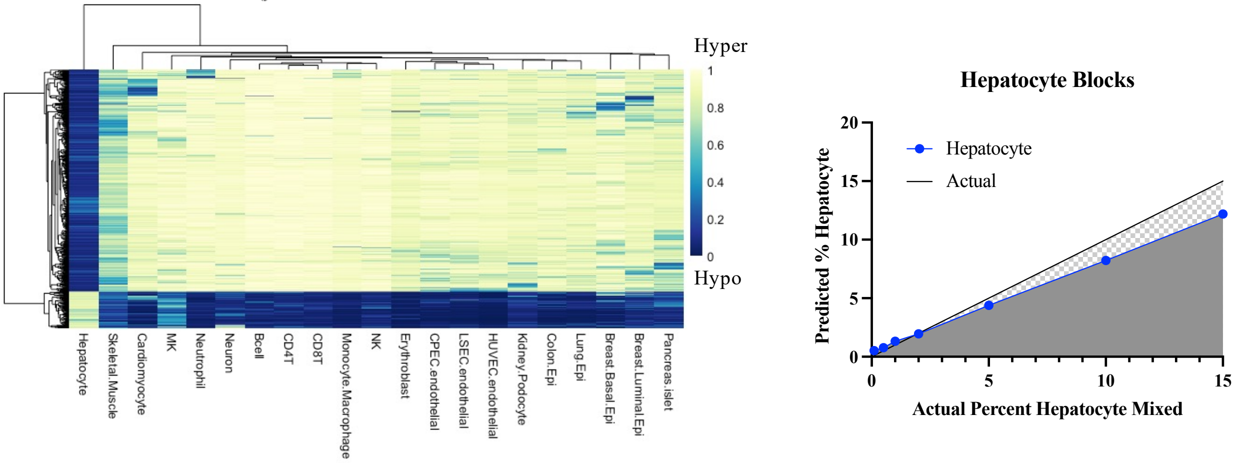


C

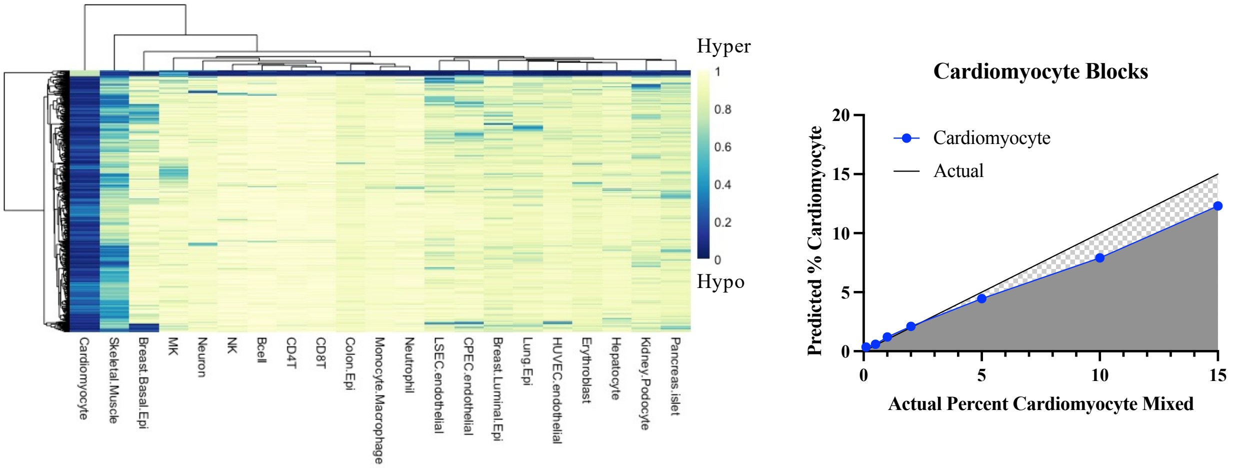


D

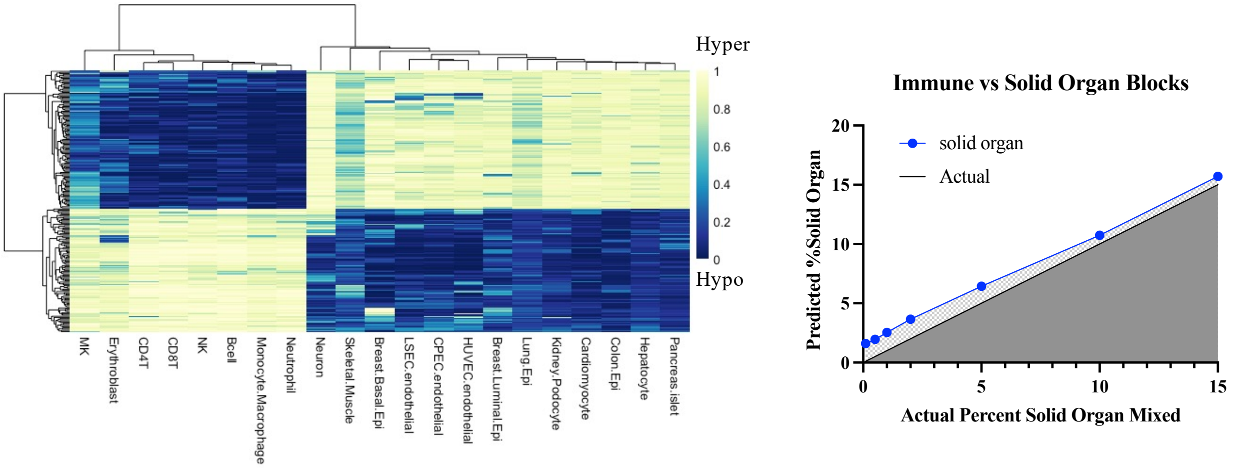

**Supplemental Figure 10. Sensitivity and specificity of identified human cell-type specific differentially methylated blocks.** (A-D) (Left) Heatmap of all cell-type specific methylation blocks selected for each target cell type. All blocks contain $\geq$3 CpG sites and have a margin of beta difference greater than or equal to 0.4 separating the target cell type from all others included in the reference maps. (Right) In-silico mix-in validation from the fragment-level probabilistic deconvolution model. Target cell-type read-pairs were in-silico mixed into a background of lymphocyte or buffy coat read-pairs at various known percentages (0.1%, 0.5%, 1%, 2%, 5%, 10%, 15%). The deconvolution model was validated on these in-silico mixed samples of known cell-type proportions at the blocks selected. The average predicted %target is graphed relative to the known %mixed to assess sensitivity and specificity of the identified cell-type specific blocks and deconvolution model. Data presented as mean ± SD; N=3 replicates per proportion.

**Supplemental Figure 11.**

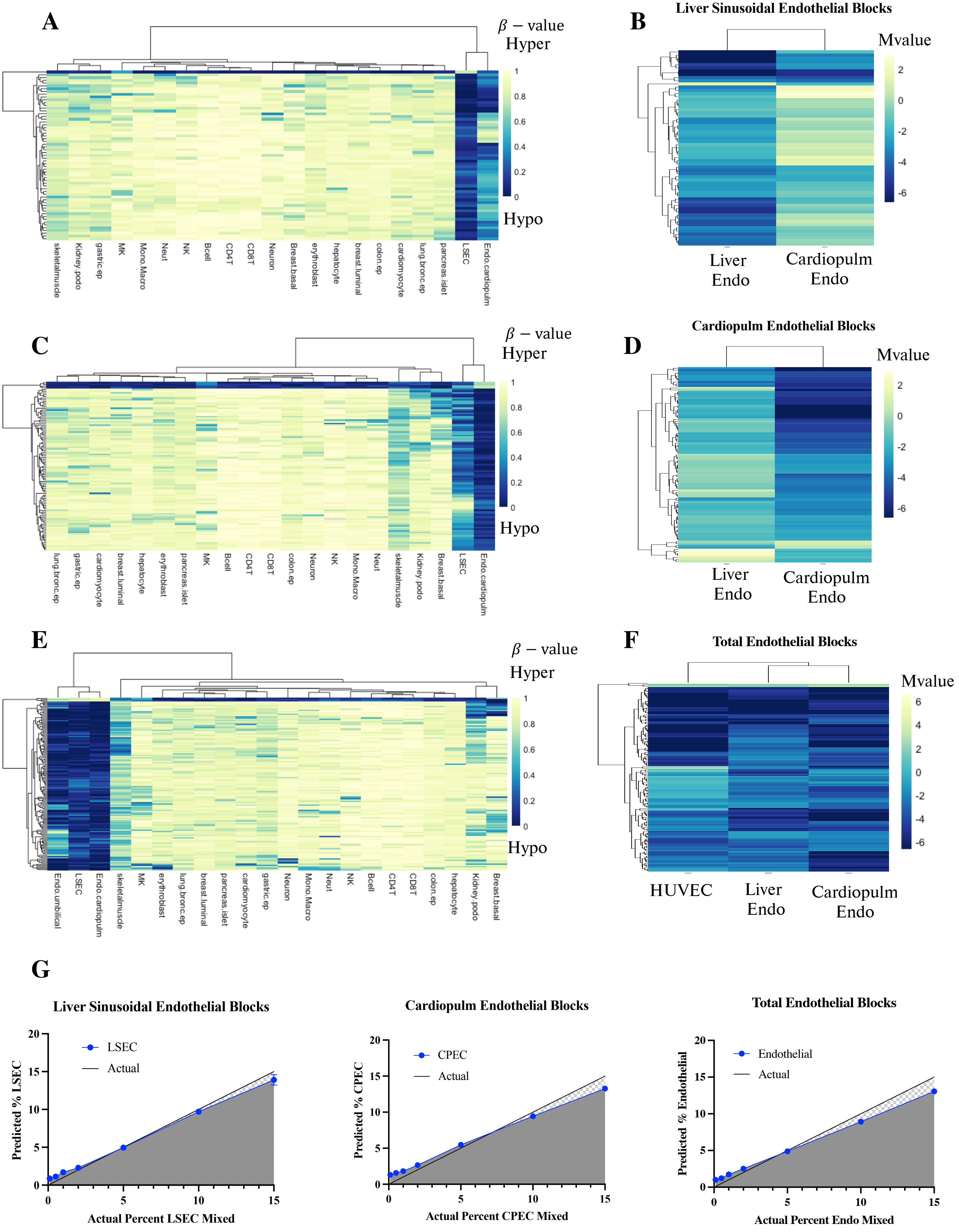

**Supplemental Figure 11. Sensitivity and specificity of identified human endothelial specific differentially methylated blocks.** (A, C, E) Heatmap of cell-type specific methylation blocks selected for each target cell type. All blocks contain $\geq$3 CpG sites and have a margin of beta difference greater than or equal to 0.4 separating the target cell type from all others included in the reference maps. (B, D, F) Enlarged heatmap of cardiopulmonary (B) or liver endothelial (D) specific methylation blocks that are unique relative to other liver or cardiopulmonary endothelial blocks respectively; (F) pan-endothelial specific methylation blocks with common methylation status amongst cardiopulmonary, liver, and HUVEC endothelial cell populations. Methylation status is represented by M-values (Logit transformation of $\beta$-values) to limit heteroscedasticity in visual representation of methylation differences across regions. (G) In-silico mix-in validation from a fragment-level probabilistic deconvolution model. Target cell-type read-pairs were in-silico mixed into a background of lymphocyte or buffy coat read-pairs at various known percentages (0.1%, 0.5%, 1%, 2%, 5%, 10%, 15%). The deconvolution model was validated on these in-silico mixed samples of known cell-type proportions at the blocks selected. The average predicted %target is graphed relative to the known %mixed to assess sensitivity and specificity of the identified cell-type specific blocks and deconvolution model. Data presented as mean ± SD; N=3 replicates per proportion.

# Supplemental Figure 12.

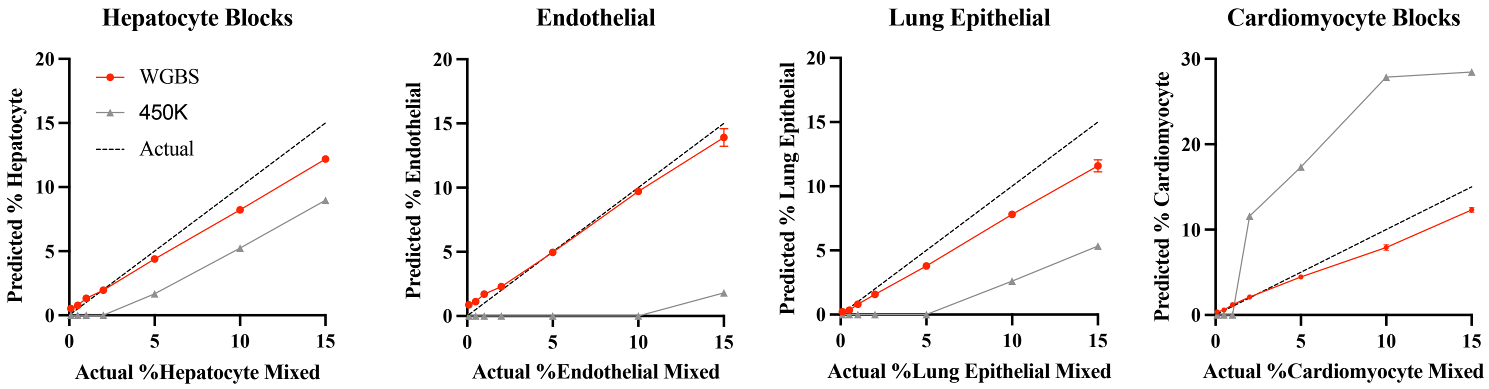

**Supplemental Figure 12. Performance of the probabilistic fragment-level deconvolution algorithm using WGBS data relative to NNLS MethAtlas from 450K array data.** Cell type specific markers outperform the array-based atlas and achieve <0.1% resolution. Shown are in silico simulations for four cell types, computationally mixed within leukocytes at various known percentages (0.05%, 0.1%, 0.5%, 1%, 2%, 5%, 10%, 15%). Each mixture was analyzed using our WGBS atlas and fragment-level deconvolution model (red), compared to Moss et al. 2018 (gray). Data presented as mean ± SD; N=3 replicates per proportion.

# Supplemental Figure 13.

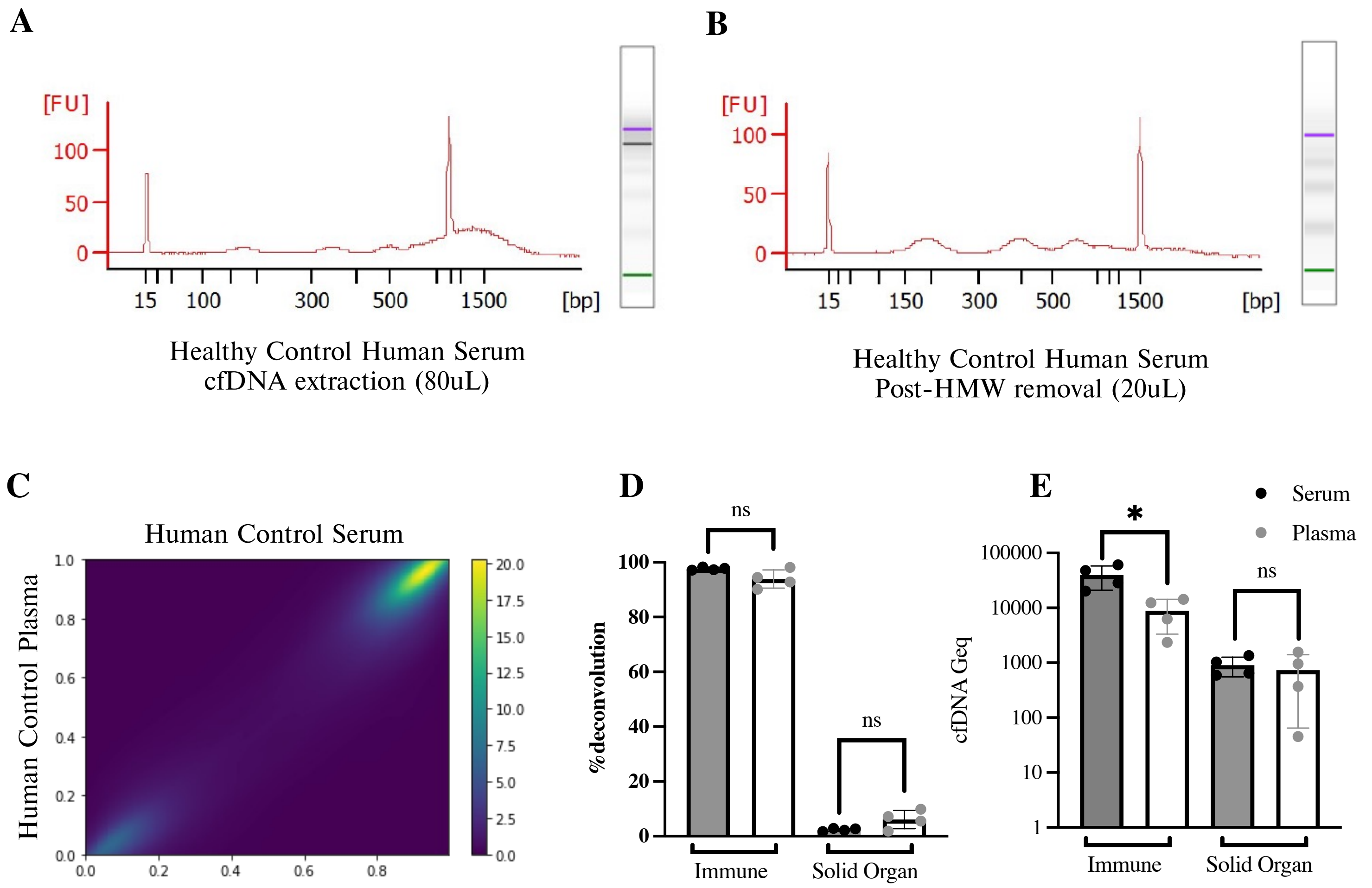

**Supplemental Figure 13. Comparison of methylation status and cellular origins of cfDNA isolated from serum and plasma of healthy human controls.** (A, B) Representative bioanalyzer trace of freshly isolated cfDNA extracted from healthy control human serum before (A) and after (B) removal of high-molecular weight (HMW) DNA. (C) Density scatter plot comparing methylation status across blocks in cfDNA isolated from control human serum (n=4) versus control human plasma (n=4). Methylation levels are highly correlated at the block-level with Pearson's r =0.95 and $R^2$=0.89. (D) Predicted %Immune versus %Solid Organ derived cfDNA extracted from either serum or plasma. Origins were assessed at n = 222 methylation blocks found to separate immune cell types from solid organ cell types. (E) Immune and solid organ Geq from cfDNA isolated from serum versus plasma. (D, E) Data presented as mean ± SD; N=4 samples per group. Mann-Whitney test was used for comparisons amongst groups. ns, $p \geq 0.05$; *, $p < 0.05$.

# Supplemental Figure 14.

A

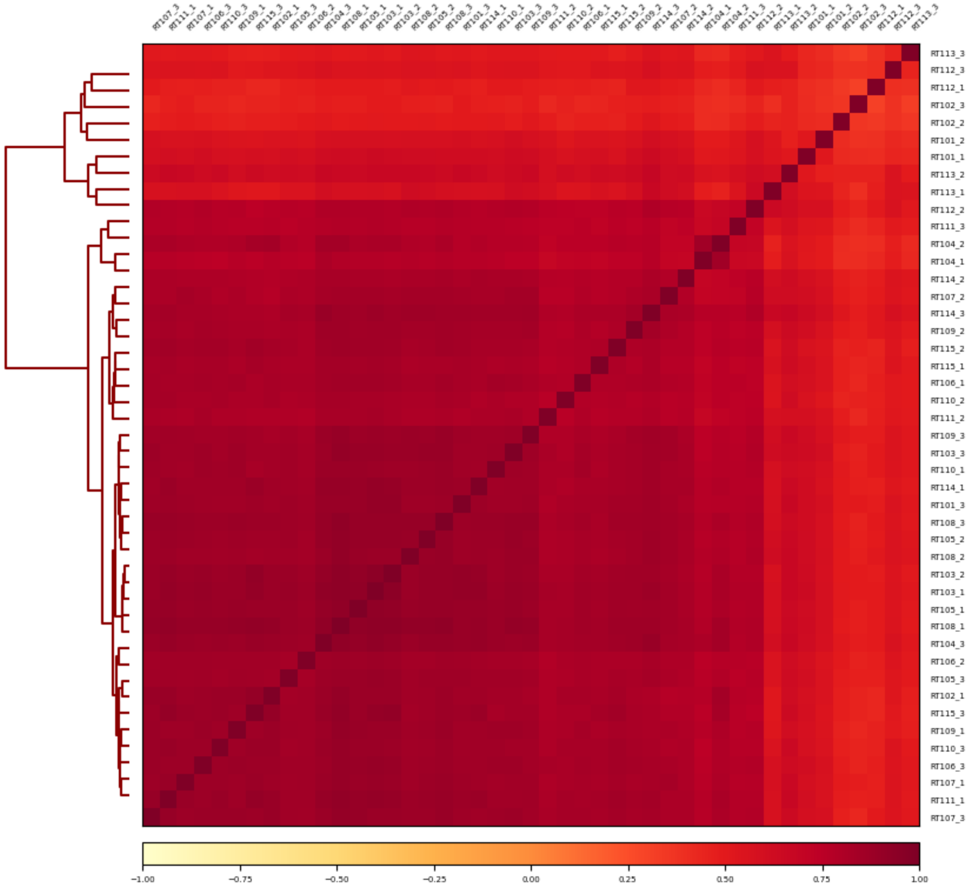


B

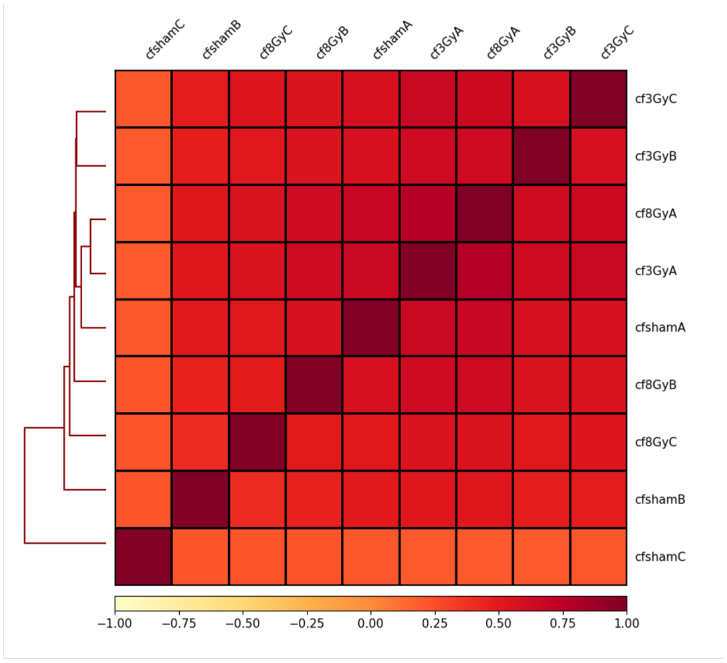

**Supplemental Figure 14. Individual sample correlation of sequencing parameters and coverage amongst cell-free DNA sequencing libraries.** Sample correlation of methylation sequencing libraries comparing distribution of aligned and filtered high-quality paired-end reads from (A) Breast Cancer cell-free DNA methylome data and (B) Mouse cell-free DNA methylome data. Correlation heatmap was generated using deepTools plotCorrelation (--corMethod spearman) based on the output of multiBamSummary collapsed over the bed file of captured probe regions.

**Supplemental Figure 15.**

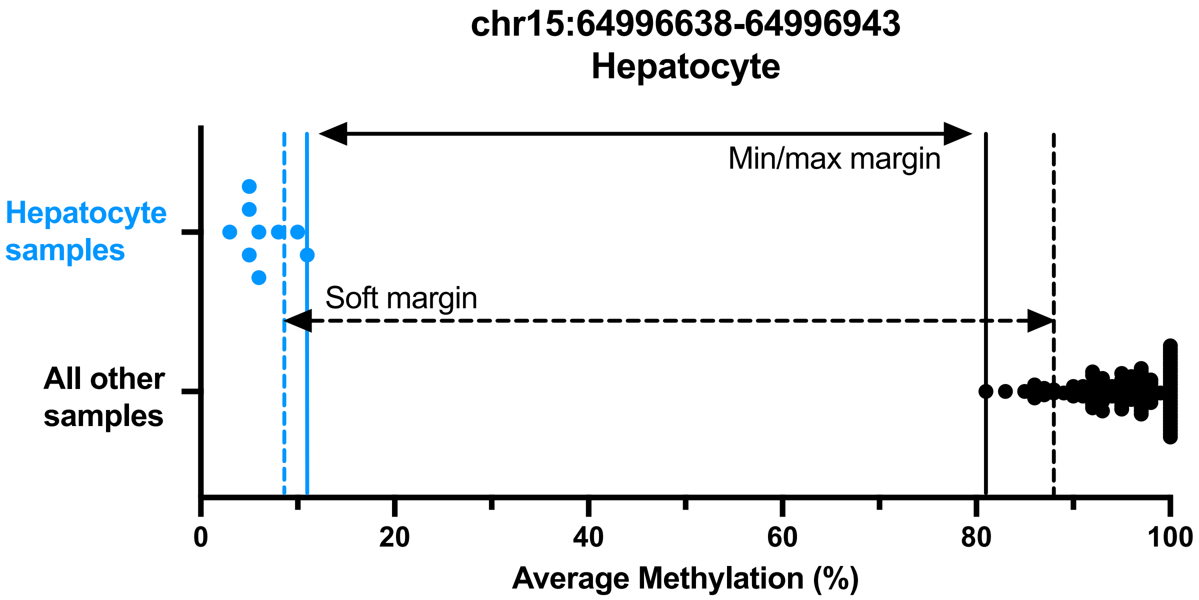

**Supplemental Figure 15. Representative diagram of the feature selection process implemented to identify cell-type specific blocks (DMBs).** Briefly, the Average Methylation Fraction (AMF) was calculated for each block in every sample (with ≥3 CpGs and length less than 2Kb). Differential blocks were sorted by the margin of separation, termed "delta-beta", defined as the minimal difference between the average methylation in any sample from the target group vs all other samples. Then, we computed the "soft margin" between target samples and background samples, allowing for some outliers using percentiles. For example, at the hepatocyte-specific DMB shown below, the AMF of individual hepatocyte samples (n=9) ranged from 0.03-0.11. In contrast, the AMF at this block for all other samples (n=95) ranged from 0.81-1.0. We calculated the difference between the 80$^{th}$ percentile of the methylation status in the target group of all hepatocyte samples (0.09) and the 10$^{th}$ percentile of the methylation status in the background group (0.88) and used this to compute the "soft margin" of separation at 0.79 that is plotted below using dotted lines. We required all cell-type specific blocks identified to have a "soft margin" ≥ 0.4 for human and ≥ 0.35 for mouse.

## Legends for Supplemental Tables

**Supplemental Table 1.** Human reference methylation data from healthy tissues and cell types.

**Supplemental Table 2.** Mouse reference methylation data from healthy tissues and cell types.

**Supplemental Table 3.** Identified human cell-type specific methylation blocks used to identify origins of cell-free DNA (margin 0.4;bg.quant 0.1). Annotation was performed using Homer. The margin of separation represents the delta-beta (maximum higher – minimum lower) across all samples. Blocks with a (-) direction are hypomethylated and (+) direction are hypermethylated. AMF (average methylation fraction) indicated as a fraction.

**Supplemental Table 4**. Identified mouse tissue and cell-type specific methylation blocks used to identify origins of cell-free DNA (margin 0.35). Annotation was performed using Homer. The margin of separation represents the delta-beta (maximum higher – minimum lower) across all samples. Blocks with a (-) direction are hypomethylated and (+) direction are hypermethylated. AMF (average methylation fraction) indicated as a fraction.

**Supplemental Table 5.** Summary of identified human (A) and mouse (B) cell-type specific methylation blocks.

**Supplemental Table 6.** Significantly enriched biological pathways and functions for genes associated with differential methylation in each cell-type.

**Supplemental Table 7.** Genomic annotation of identified human and mouse cell-type specific hypomethylated and hypermethylated blocks relative to all captured blocks.

**Supplemental Table 8.** Extended endothelial-specific methylation blocks (margin 0.4;bg.quant 0.2) used for pathway analysis and validation of cell identity through integration with paired RNA expression data.

**Supplemental Table 9.** Primers used for RT–qPCR in radiation-treated mouse tissues.

**Supplemental Table 10.** Characteristics of breast cancer patients enrolled in this study. Individual patient characteristics described in Supplemental Table 11.

**Supplemental Table 11.** Extended clinical data and characteristics of individual breast cancer patients enrolled in this study.

**Supplemental Table 12.** Mouse cfDNA sample concentrations and predicted cell-type

proportions from deconvolution analysis at identified cell-type specific blocks for target cell types.

**Supplemental Table 13.** Human cfDNA sample concentrations and predicted cell-type proportions from deconvolution analysis at identified cell-type specific blocks for target cell types.

**Supplemental Table 14.** Cross-species comparison of cell-type specific DMBs (differentially methylated blocks) in human and mouse species.

**Supplemental Table 15.** Extended immune and endothelial-specific methylation blocks (margin 0.4;bg.quant 0.1). from purified cell populations that were not used to identify origins of cell-free DNA.
